# Unravelling the Genetic Mechanisms of Litter Traits in a Maternal Line of Pigs

**DOI:** 10.1111/jbg.70042

**Published:** 2026-02-12

**Authors:** Guilherme Oselame, Suelen Fernandes Padilha, Maurício Egídio Cantão, Jane de Oliveira Peixoto, Aline Zampar, Adriana Mércia Guaratini Ibelli, Luis Orlando Duitama Carreño, Jader Silva Lopes, Pedro Henrique Ferreira Freitas, Marcelo Silva Freitas, Mônica Corrêa Ledur

**Affiliations:** ^1^ Programa de Pós‐Graduação Em Zootecnia Universidade Do Estado de Santa Catarina (UDESC Oeste) Chapecó Santa Catarina Brazil; ^2^ Programa de Pós‐Graduação Em Zootecnia Universidade Federal Do Paraná Curitiba Paraná Brazil; ^3^ Embrapa Suínos e Aves Concórdia Santa Catarina Brazil; ^4^ Embrapa Pecuária Sudeste São Carlos São Paulo Brazil; ^5^ BRF SA Curitiba Paraná Brazil

**Keywords:** animal breeding, candidate genes, genetic parameters, GWAS, reproductive efficiency, swine

## Abstract

Reproductive traits related to litter size are the main indicators of reproductive efficiency in pig production and are continuously evaluated for the selection of maternal lines. Several environmental and genetic factors are involved with the development of these traits. Genome‐wide association studies (GWAS) allow a better understanding of the genetic control of complex traits, especially those with low heritability (*h* ^2^). Therefore, this study aims to estimate the genetic parameters and to identify genomic regions and candidate genes associated with total number born (TNB), number born alive (NBA), and viable piglets at Day 5 (PV5) in a Large White female line. For this, 17,011 phenotypic records, 190,000 pedigree records, and 4366 animals genotyped with the Illumina 50 K and 80 K panels were used. Estimates of *h* ^2^, genetic (*r*_*g*_) and phenotypic (*r*_*p*_) correlations, and GWAS were performed with the BLUPF90 family programs. Positional candidate genes, their main biological processes, and networks were investigated using the Ensembl database and the BioMart, PANTHERdb, and STRING tools. The studied traits presented low *h* ^2^ estimates, but with high and positive *r*_*g*_ and *r*_*p*_. In the GWAS, 14 significant genomic windows were identified for TNB, 10 for NBA, and 15 for PV5. These regions include 157 genes for TNB, 101 for NBA, and 140 for PV5, mapped across 10 different chromosomes. Among the genes located in those regions, the *ESR1, THRB, SLIT2*, and *ZBTB2* were common to the three traits and are involved in processes of hormonal regulation, embryogenesis, immunity, and homeostasis. Moreover, 12 of those genes were new positional candidates for TNB, NBA, or PV5. Among them, we highlight the *FSTL4*, *PAPPA*, and *TCF7* genes associated with PV5, which are involved with hormonal regulation, growth factors, and immunity, respectively. The *SLIT2*, *MTHFD1L*, *OVOL2*, *SHB*, and *EXOSC3* genes, involved with embryogenesis and neurogenesis, were associated with TNB and NBA. Furthermore, uncharacterized genes, such as *ENSSSCG00000058091*, related to mitochondrial homeostasis, were associated with TNB, while *ENSSSCG00000040472*, related to protein synthesis, was identified for PV5. These new findings reveal common and exclusive genetic mechanisms that may influence important litter traits in pigs, helping the development of breeding strategies to optimise reproductive efficiency.

## 1 Introduction

The understanding of the genetic parameters for reproductive traits is crucial, as females must have a high farrowing rate but also ensure that their offspring reach at the weaning age (Kobek‐Kjeldager et al. 2020). Among reproductive traits, those related to litter size, such as total number born (TNB), number born alive (NBA), and viable piglets at Day 5 (PV5), hold significant economic value in pig production. However, these traits are directly influenced by environmental factors and not just by genetic effects, having low heritability (*h* ^2^) (Holm et al. 2004; Ogawa et al. 2018). Moreover, complex interactions exist between maternal additive genetic effects and direct additive genetic effects of piglets, which presents a complicating factor for obtaining genetic gains aimed at increasing the number of live‐born piglets while simultaneously reducing mortality rates (Alves 2018).

Several studies have estimated the genetic parameters for TNB and NBA (Nielsen et al. 2013; Su 2020; Kramer et al. 2021; Sell‐Kubiak 2021). Generally, a wide range of *h* ^2^ estimates has been reported for different genetic groups, from 0.06 ± 0.00 to 0.19 ± 0.00, although all values remain of low magnitude (Nielsen et al. 2013; Ogawa et al. 2018; Su et al. 2007; Kramer et al. 2021). For PV5, there is relatively scarce information, with some results reported by Su et al. (2007), Su et al. (2008), Nielsen et al. (2013), Putz et al. (2015), and Su (2020), ranging from 0.01 to 0.11 ± 0.02, all of those of low magnitude.

For genetic (*r*_*g*_) and phenotypic (*r*_*p*_) correlations, TNB and NBA typically show high positive correlations, ranging from 0.60 ± 0.01 to 0.99 ± 0.01 (Roehe and Kennedy 1995; Su et al. 2007; Zhang et al. 2019; Sell‐Kubiak 2021). In contrast, PV5 and TNB or NBA exhibit great variability. Estimates of r_g_ between LV5 and TNB were positive and moderate (0.34 ± 0.11) to high (0.85 ± 0.02) (Su et al. 2007; Nielsen et al. 2013; Putz et al. 2015), and the *r*_*g*_ between PV5 and NBA was high, ranging from 0.57 ± 0.12 to 0.83 ± 0.00 (Su et al. 2007; Putz et al. 2015). Thus, in addition to the significant environmental influence for these traits, genetic parameters also vary depending on the breed and population evaluated (Holm et al. 2004; Zhang et al. 2015; Yu et al. 2022).

The integration of genomic, pedigree, and phenotypic data enhances the model's robustness, thereby improving the estimation of genetic parameters (VanRaden 2007). For genomic association analyses, single nucleotide polymorphisms (SNPs) are primarily utilized as genetic markers (Dekkers 2012). The reduced cost of genotyping SNP panels has enabled the implementation of genomic selection, which has led to greater genetic gains in breeding programs (Cleveland and Hickey 2013). Furthermore, these panels have made it easier to conduct genome‐wide association studies (GWAS), improving our knowledge of how genetics influence complex traits (Uzzaman et al. 2018; Liu et al. 2022).

In swine, GWAS methodology has enabled the identification of genomic regions associated with traits of interest (Dekkers 2012; Martins et al. 2022; Sun et al. 2023). Over the years, several genomic regions and genes have been associated with reproductive traits in pigs (Uzzaman et al. 2018; Liu et al. 2022; Sun et al. 2023; Hong et al. 2024). Some examples include genes associated with gestation length (Sun et al. 2023), total born, born alive, and weaned piglets (Uzzaman et al. 2018), litter size (Hong et al. 2024), and other traits closely related to maternal capacity in sows, such as teat number (Liu et al. 2022).

The differences between pig lines and breeds become more evident when genomic studies are conducted. Jiang et al. (2019) performed a GWAS for TNB and NBA using four Yorkshire (also known as Large White) swine populations, two of which exhibited no genetic differences between them in the principal component analysis. Nevertheless, the association analysis revealed distinct positional candidate genes in each of those populations. Although genomic studies related to PV5 are limited, Guo et al. (2016) evaluated the traits of PV5 and mortality rate before day 5 (MORT) in Landrace and Yorkshire pigs. In the Landrace population, four quantitative trait loci (QTL) regions on *Sus Scrofa* chromosome (SSC) 6, 7, 13, and 14 were identified for PV5, and two regions (SSC 2 and 7) for MORT. In contrast, only one QTL region on SSC9 was associated with PV5 in the Yorkshire population (Guo et al. 2016).

Thus, the GWAS conducted across different populations expands the possibility of finding potential genetic markers associated with the traits, in addition to identifying positional candidate genes within specific lines. Therefore, this study aimed to estimate genetic parameters for TNB, NBA, and PV5 traits and to identify genomic regions and candidate genes associated with the development of these traits in a Large White maternal pure line of pigs.

## 2 Material and Methods

All methods and procedures used in this study were reviewed and approved by the Ethics Committee on Animal Use (CEUA) from Embrapa Swine and Poultry National Research Center, under protocol # 002/2016, in agreement with the rules established by the National Council of Animal Experimentation Control (CONCEA) to ensure compliance with international guidelines for animal welfare.

The data used in this study were obtained from a nucleus farm from the Genetic Breeding Program of the BRF S.A. Company, located in the western region of the Santa Catarina State, in southern Brazil. The study utilised data of purebred Large White animals from a maternal line, considering: (1) phenotypic information, (2) pedigree records (animal, sire, and dam), and (3) genotypic data.

### 2.1 Phenotype, Genotype, and Pedigree Information

The pedigree database contained 190,428 records of animals, sires, and dams born between 2001 and 2024. The phenotypic information consisted of 17,011 records from the same period, including data on birthdate of replacement females, mating date, farrowing date (when applicable), farrowing order (FO) of pregnant females, number of mummified piglets, stillborns, live‐born piglets, total born piglets, and viable piglets at Day 5.

The genotyped animal data comprised 4366 records of males and females collected between 2010 and 2023. The DNA was extracted from tail tissue, collected during routine tail docking procedures, and stored in the company's tissue bank. Animals were genotyped using the GGP Porcine 50 K BeadChip and Porcine SNP80 BeadChip, both from Illumina (San Diego, CA, USA). For the genetic parameter estimates and GWAS analyses, a total of 47,680 common SNPs in both BeadChips were used.

### 2.2 Phenotypic Data Quality Control

Of the 17,011 phenotypic data collected from 2001 to 2024, only records from 2009 to 2024 were used, and sows without farrowing records were excluded. Outlier removal followed a sequential chronological order: first applied to TNB, then NBA, and finally to PV5. All missing records for TNB were excluded, followed by the removal of data exceeding ±3 standard deviations (SD) from the mean for each trait. The means considered were 13.35 ± 3.60 piglets for TNB, 12.07 ± 3.29 for NBA, and 10.67 ± 2.93 piglets for PV5.

Since selection in the nucleus farm occurs weekly, contemporary groups (CG) were defined based on the year and week of farrowing, including only sows with up to six parities. This definition implicitly accounts for environmental variations such as seasonality, temperature fluctuations, and transient management changes. For further analysis, only CG with 10 or more animals were retained. After quality control (QC), 8266 farrowing records remained distributed across 196 CGs. The final phenotypic data comprised 32.7% first parity sows, 33.1% second parity, 19.3% third, 11.2% fourth, 3.2% fifth and 0.5% sixth parity sows. The pedigree‐based relationship matrix comprised 3482 animals, and the genomic relationship matrix included 4366 animals.

The environmental factors influencing the evaluated traits were tested in an analysis of variance (ANOVA) performed using the Generalised linear model (GLM) procedure from R software version 4.2.2 (R Core Team, 2022). The fixed effects of CG and FO were significant; therefore, they were used in further analysis.

### 2.3 Genotypes and Samples Data Quality Control

The QC of genotyped samples and SNPs was performed using the PREGSF90 package from the BLUPF90 program family (Lourenco et al. 2022), according to the following criteria: SNPs with Hardy–Weinberg equilibrium < 0.15, minor allele frequency < 0.02, SNP call rate < 0.98, and sample call rate < 0.90 were removed. Additionally, animals that showed inconsistencies in the relationship matrix, non‐autosomal SNPs, and SNPs without a defined genome position were also removed. From the 47,680 SNPs and 4366 samples, after QC, 35,357 SNPs and 4337 animals remained for further analysis.

### 2.4 Estimation of Genetic Parameters

The genetic parameter estimates were conducted using a multi‐trait animal model, including the three traits: TNB, NBA, and PV5. This approach was chosen to account for the genetic correlation between traits, shown to improve accuracy and reduce bias (van der Werf 2003). The following animal model was used to estimate the variance components:

$$y_{i}=X_{i}b_{i}+Z_{i}a_{i}+W_{i}p_{i}+e_{i}$$

where $y_{i}$ is a vector of observations for the *i*‐th trait (TNB, NBA and PV5), $b_{i}$ is a vector of fixed effects (CG and FO), $a_{i}$ is the vector of random additive genetic effects ($u_{i}$ for marker‐based estimates), $p_{i}$ is the vector of permanent environmental effect, $X_{i}$, $Z_{i}$ and $W_{i}$ are the incidence matrices associated with each effect ($b_{i}$, $a_{i}$/$u_{i}$, and $p_{i}$, respectively), and $e_{i}$ is the vector of residual effects for the *i*‐th trait.

The analyses to estimate variance components were performed using BLUPF90+ with AI‐REML (Average Information Restricted Maximum Likelihood) option (Lourenco et al. 2022) in a three‐trait model (TNB, NBA, PV5). For the pedigree‐based analysis, the following assumptions were adopted: **a** ∼ N (**0**, **A** ⊗ **G**_0_), where **G**_**0**_ is the additive genetic (co)variance matrix; **p** ∼ N (**0**, **I** ⊗ **P**_0_), where **P**_0_ is the permanent environmental (co)variance matrix; and **e** ∼ N (**0**, **I** ⊗ **R**_0_), where **R**_0_ contains the residual (co)variances for the three traits, **A** is the pedigree‐based relationship matrix, and **I** is an identity matrix. For genomic‐based estimates, matrix **A** was replaced by the **H** matrix, which integrates SNP marker information with classical pedigree data (Aguilar et al. 2010). The inverse of matrix **H** is defined as follows:

$$H^{-1}=A^{-1}+\left[\begin{array}{cc} 0 & 0\\ 0 & G^{-1}-A_{22}^{-1} \end{array}\right]$$

where **A** ^−1^ represents the inverse of the pedigree relationship matrix, **G** ^−1^ is the inverse of the genomic relationship matrix, and **A**_**22**_^−1^ is the inverse of the pedigree relationship matrix for genotyped animals. For this, it was assumed that **H** ∼ N (**0**, **I** ⊗ **H**_0_), where **H**_**0**_ is the relationship matrix derived from pedigree and SNP markers.

### 2.5 Genome‐Wide Association Study

The GWAS analyses were conducted using the BLUPF90 program family (Lourenco et al. 2022) with the single‐step genomic BLUP (ssGBLUP) methodology. The genetic and residual variance components used in the analysis were estimated via a single‐trait model in the BLUPF90+ package using the AI‐REML method (Lourenco et al. 2022). The association analysis was performed using the POSTGSF90 package (Aguilar et al. 2014) with the following model:

y=Xb+Zu+Wp+e

where y is the vector of phenotypic observations, b is the vector of fixed effects (CG and FO), u is the vector of random additive genetic effects with markers, p is the vector of permanent environmental effects, X, Z and W are the incidence matrices associated with each effect (b, u and p, respectively), and e is the vector of residual effects for each phenotype. SNP effects were estimated using the ssGWAS methodology proposed by Wang et al. (2012), where 𝑚̂ = 𝐷𝑀′ [𝑀𝐷𝑀′]^−1^ â𝑔, with 𝑚̂ representing the vector of SNP effects, **D** is a diagonal matrix of SNP weights, **M** is the genotype matrix for each gene locus, and **â𝑔** is the vector of estimated genomic breeding values. In this study, equal weights were assigned to SNPs, and the proportion of genetic variance explained by each 1 Mb genomic window was estimated based on the effects of the SNPs within that window using the POSTGSF90 package (Aguilar et al. 2014), according to the following equation:

$$\frac{\mathrm{var}\left(u_{i}\right)}{\sigma_{u}^{2}}\times 100=\frac{\mathrm{var}\left(\sum_{j=1}^{N}M_{j}m_{j}\right)}{\sigma_{u}^{2}}\times 100$$

where *u*_*i*_ is the genetic value of the *i*‐th genomic region under consideration, *N* is the total number of adjacent SNP markers within the identified 1 Mb genomic region, and *m*_*j*_ is the marker effect of the *j*‐th SNP within the *i*‐th 1 Mb genomic region. For the GWAS, the genome was partitioned into non‐overlapping 1 Mb windows. This window size was defined based on the density of the SNP panel used and the linkage disequilibrium (LD) decay calculated for our population (Supporting Information, Figure S1), which indicates that LD extends over approximately 1 Mb. Moreover, this window size is generally used in variance‐based GWAS found in the literature with pigs (Schneider et al. 2015; Tang et al. 2019; Cheng et al. 2021; Padilha et al. 2025), because it can capture the heterogeneity pattern of LD in the pig genome.

The significance threshold was defined based on the expectation of an equal genetic variance explained per window, as described by Onteru et al. (2013) and Moreira et al. (2018). The expected genetic variance (EGV) was calculated as 100% of the genetic variance (GV) divided by the total number of windows identified per trait in the GWAS. Windows explained more than 10 times the EGV for the trait were considered significant (EGV = 100% VG/number of windows ×10).

### 2.6 Candidate Gene Identification and Enrichment Analysis

Once the identification of the significant windows' start and end positions for TNB, NBA, and PV5 traits, the genes located within these windows were investigated using the Ensembl BioMart tool version 113 (Kinsella et al. 2011). Genes were annotated according to the Sscrofa 11.1 genome assembly. Biological processes (BP) associated with genes in significant regions were obtained using Ensembl BioMart version 113, focusing on those potentially related to the studied traits. A functional enrichment of those BP was performed using PantherDB version 19.0 (Thomas 2021). Based on the BP (*p* < 0.05), REVIGO (Supek et al. 2011) was used to summarise BP by removing redundant GO terms.

A gene network and protein–protein interaction analyses were conducted using the online tool Network Analyst (Zhou et al. 2019) with the STRING database, using the human information (Szklarczyk et al. 2019), since there is no swine information in this database.

Finally, genes within significant windows and those highlighted in the functional enrichment analyses were cross‐referenced with public databases (NCBI: Sayers et al. 2021; OMIM: Amberger et al. 2018) and subjected to a detailed literature review to prioritise candidate genes involved with TNB, NBA, and PV5.

## 3 Results

The descriptive statistics for the evaluated traits are shown in Table 1. From 2009 to 2024, there were 8266 farrowings. The means and standard deviations (SD) for the TNB, NBA, and PV5 traits were 13.35 (3.60) for TNB, 12.07 (3.29) for NBA and 10.67 (2.93) for PV5 (Table 1). The effects of CG and FO were significant (*p* < 0.001) in the analysis of variance for the evaluated traits and were therefore included in the model for estimating the genetic parameters and in the GWAS analysis.

**TABLE 1:** Means, standard deviation, coefficient of variation (CV), minimum, and maximum values for the three litter traits evaluated.

Traits	Mean	Standard deviation	CV (%)	Minimum	Maximum
TNB	13.35	3.60	26.96	2	24
NBA	12.07	3.29	27.31	1	21
PV5	10.67	2.93	27.52	1	19

Abbreviations: NBA, number born alive; PV5, viable piglets at Day 5; TNB, total number born.

### 3.1 Variance Components, Heritability, Genetic and Phenotypic Correlations

The additive genetic and permanent environmental variance components estimated for TNB, NBA and PV5 through a multi‐trait animal model showed slight differences between the pedigree and genomic‐based methods (Table 2).

**TABLE 2:** Variance components for the three traits estimated through a multi‐trait model using pedigree and genomics‐based methods. Analyses performed with the BLUPF90 family programs.

Traits	Pedigree method			Genomic method		
	$\sigma_{a}^{2}$	$\sigma_{p}^{2}$	$\sigma_{\mathrm{pe}}^{2}$	$\sigma_{a}^{2}$	$\sigma_{p}^{2}$	$\sigma_{\mathrm{pe}}^{2}$
TNB	1.76	9.74	0.69	1.64	9.74	0.79
NBA	1.46	8.66	0.45	1.30	8.66	0.59
PV5	0.98	6.96	0.45	0.85	6.96	0.59

Abbreviations: $\sigma_{a}^{2}$, additive genetic variance; $\sigma_{p}^{2}$, phenotypic variance; $\sigma_{\mathrm{pe}}^{2}$, permanent environmental variance; NBA, number born alive; PV5, viable piglets at Day 5; TNB, total number born.

When estimated based on pedigree (3 generations), there was an increase in $\sigma_{a}^{2}$ and a reduction in $\sigma_{\mathrm{pe}}^{2}$ compared to genomic‐based estimates. Similar results were obtained when these variance components were estimated using single‐trait models integrating pedigree and genomic information for subsequent use in the GWAS analyses (Table 3).

**TABLE 3:** Variance components for the three traits, estimated using integrated pedigree and genomic information in a single‐trait model. Analyses performed with the BLUPF90 family programs.

Traits	$\sigma_{a}^{2}$	$\sigma_{p}^{2}$	$\sigma_{\mathrm{pe}}^{2}$
TNB	1.67	9.74	0.77
NBA	1.34	8.66	0.57
PV5	0.84	6.96	0.60

Abbreviations: $\sigma_{a}^{2}$, additive genetic variance; $\sigma_{p}^{2}$, phenotypic variance; $\sigma_{\mathrm{pe}}^{2}$, permanent environmental variance; NBA, number born alive; PV5, viable piglets at Day 5; TNB, total number born.

The *h* ^2^ estimates derived from both pedigree‐based and genomic information using the multi‐trait variance components were similar for all evaluated traits, ranging from 0.11 ± 0.01 to 0.14 ± 0.01 for pedigree and from 0.10 ± 0.01 to 0.13 ± 0.01 for genomic‐based estimates (Table 4). The obtained *h* ^2^ estimates were of low magnitude, indicating a great influence of environmental variance compared to the additive genetic variance, especially for PV5.

**TABLE 4:** Heritability (*h* ^2^) estimates, genetic (*r*_*g*_), and phenotypic correlations (*r*_*p*_) for the three traits with their respective standard deviations (SD), using pedigree and genomic‐based methods. Analyses performed with the BLUPF90 family programs.

Traits	Pedigree method			Genomic method		
	TNB	NBA	PV5	TNB	NBA	PV5
TNB	**0.14 ± 0.01**	0.94 ± 0.01	0.74 ± 0.05	**0.13 ± 0.01**	0.94 ± 0.01	0.76 ± 0.04
NBA	0.90 ± 0.002	**0.13 ± 0.01**	0.82 ± 0.03	0.90 ± 0.002	**0.12 ± 0.01**	0.83 ± 0.03
PV5	0.73 ± 0.005	0.84 ± 0.03	**0.11 ± 0.01**	0.74 ± 0.005	0.85 ± 0.003	**0.10 ± 0.01**

*Note:* The *h* ^2^ estimates are on the diagonal in bold, *r*_*g*_ are above, and *r*_*p*_ estimates are below the diagonal.

Abbreviations: NBA, number born alive; PV5, viable piglets at Day 5; TNB, total number born.

Genetic and phenotypic correlation between pedigree‐ and genomic‐based estimates were also similar for TNB, NBA and PV5 (Table 4). The *r*_*g*_ and *r*_*p*_ between traits were high and positive, indicating that selection for a trait will possibly result in correlated responses to the other traits. The correlation coefficients between TNB and NBA were higher than that between these traits and PV5 (Table 4).

### 3.2 Genomic Regions and Candidate Genes

**FIGURE 1:**
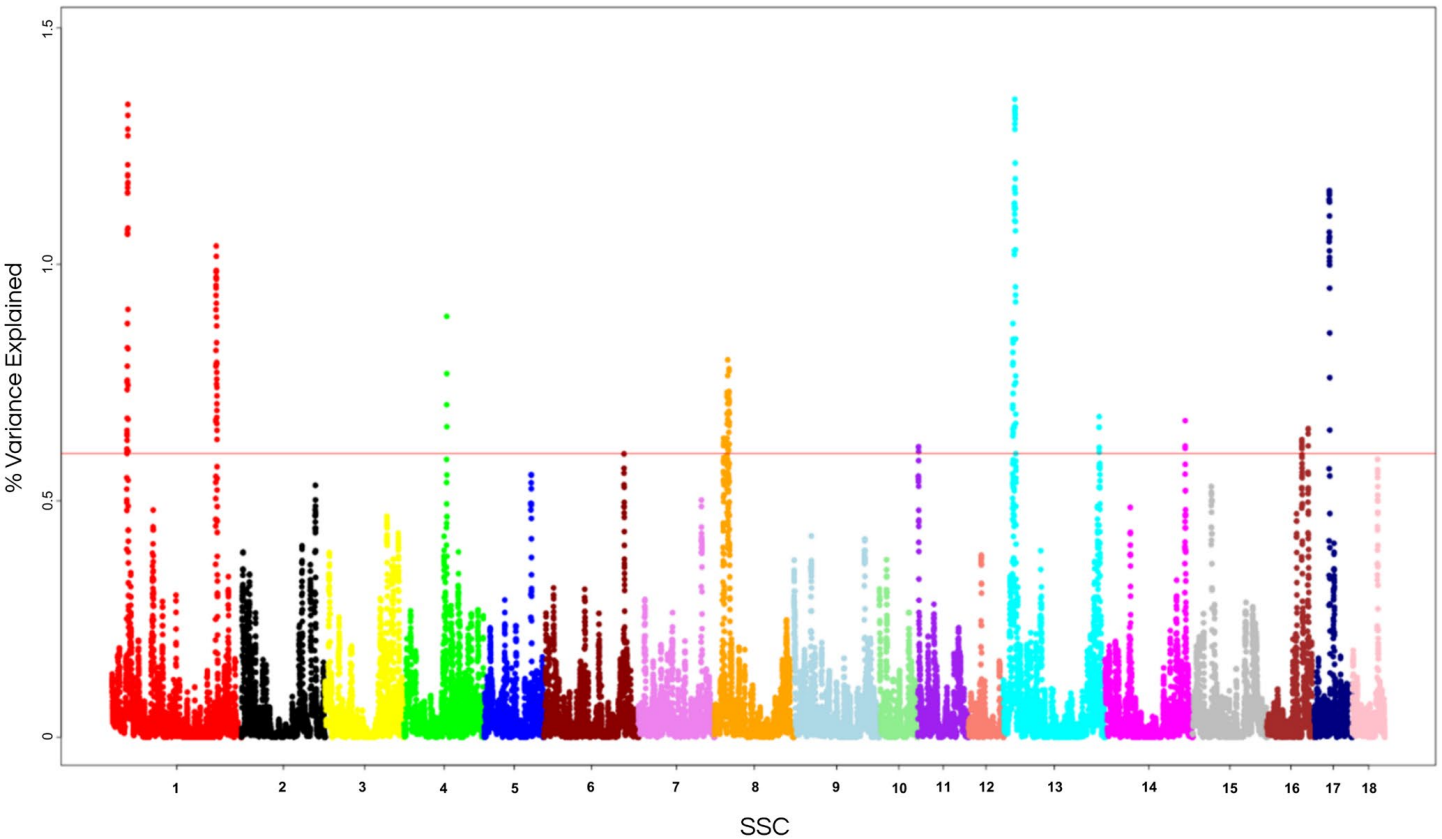
Manhattan plot of the percentage of genetic variance for total number born (TNB) explained by 1 Mb windows. The red line indicates the significance threshold (0.60%). Analysis performed using the BLUPF90 family programs and the Manhattan plot was constructed with R software version 4.2.2. [Colour figure can be viewed at wileyonlinelibrary.com]

**FIGURE 2:**
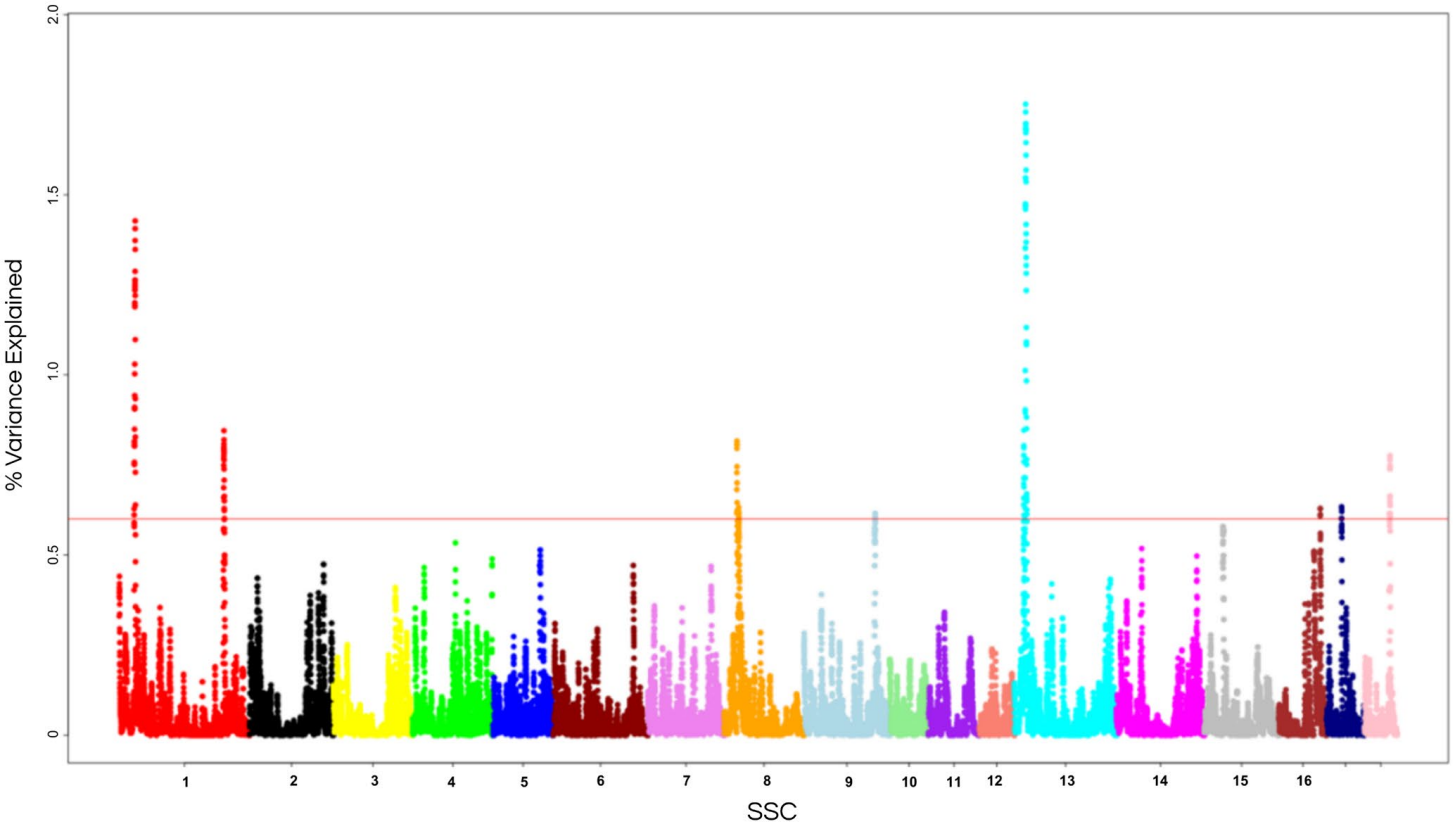
Manhattan plot of the percentage of genetic variance for Number Born Alive (NBA) explained by 1‐Mb windows. The red line indicates the significance threshold (0.60%). Analysis performed using the BLUPF90 family programs and the Manhattan plot was constructed with R software version 4.2.2. [Colour figure can be viewed at wileyonlinelibrary.com]

**FIGURE 3:**
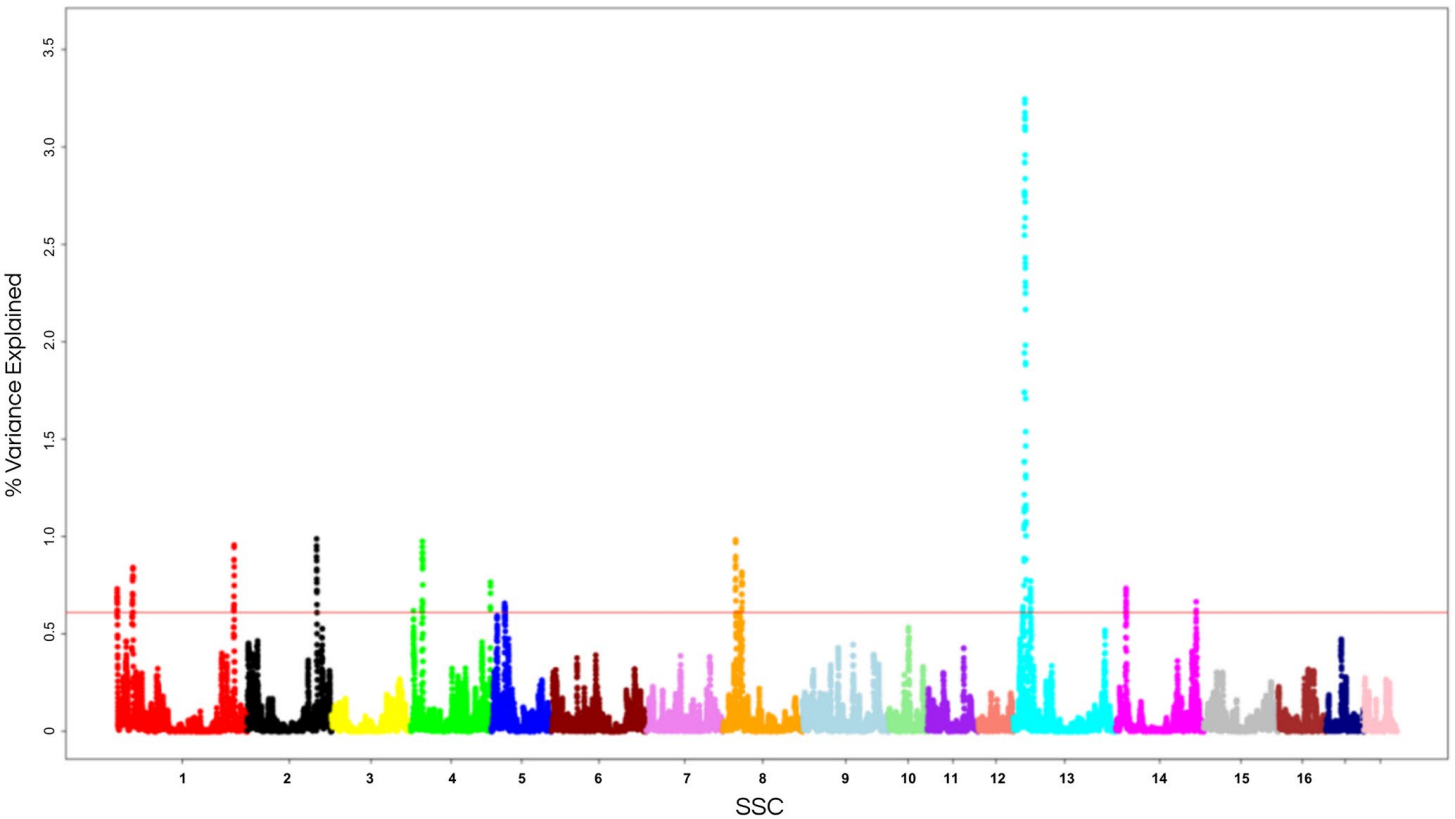
Manhattan plot of the percentage of genetic variance for Viable Piglets at Day 5 (PV5) explained by 1‐Mb windows. The red line indicates the significance threshold (0.61%). Analysis performed using the BLUPF90 family programs and the Manhattan plot was constructed with R software version 4.2.2. [Colour figure can be viewed at wileyonlinelibrary.com]

In the GWAS analysis, 1649 1‐Mb windows were identified for TNB, 1662 for NBA, and 1632 for PV5. The threshold for TNB was 0.60% (100%/1649 windows ×10), selecting windows that explained more than 10 times the expected genetic variance for this trait. Thus, 14 windows were associated with TNB, located on SSC1, 4, 8, 11, 13, 14, 16, and 17 (Figure 1), with window variances ranging from 0.61% to 1.34%, and 157 genes identified in these regions (Supporting Information, Table S1). For NBA, the threshold was also 0.60% (100%/1662 windows ×10), with 10 significant windows found on SSC1, 8, 9, 13, 16, 17, and 18 (Figure 2) explaining 0.61% to 1.75% of the genetic variance, comprising 101 genes (Supporting Information, Table S1). For PV5, the threshold was 0.61% (100%/1632 windows ×10), with 15 significant windows identified on SSC 1, 8, 9, 13, 16, 17, and 18 (Figure 3), explaining 0.61% to 3.24% of the genetic variance, where 140 genes were located (Supporting Information, Table S1). All together, these windows explained 12.09%, 8.97%, and 14.38% of the total additive genetic variance for TNB, NBA, and PV5, respectively.

The findings for TNB, NBA, and PV5 exhibited some shared regions (Figures 1, 2, 3), particularly in the windows explaining the highest genetic variance across all three traits, as in SSC13 (1.34%, 1.75%, and 3.24% for TNB, NBA, and PV5, respectively), in which the *THRB* gene is a common candidate for all evaluated traits (Table 5).

**TABLE 5:** Characterisation of genomic windows explaining more than 0.90% of the genetic variance for the three evaluated traits.

Traits	SSC	Start position (bp)	End position (bp)	Variance explained (%)	Genes in the windows
TNB	13	11,126,421	12,109,287	1.34%	*THRB*
TNB	1	14,239,508	15,228,370	1.33%	*ESR1, CCDC170, ARMT1, RMND1, ZBTB2, AKAP12, MTHFD1L e ENSSSCG00000046251*
TNB	17	26,396,683	27,371,093	1.15%	*OVOL2, PET117, KAT14, ZNF133, DZANK1, POLR3F, RBBP9, SEC23B, SMIM26, DTD1, SCP2D1 e SLC24A3*
TNB	1	237,779,701	238,754,516	1.03%	*PAX5, ZCCHC7, GRHPR, ZBTB5, POLR1E, FBXO10, FRMPD1, TRMT10B, EXOSC3, DCAF10, SHB, SLC25A51 e ENSSSCG00000058091*
NBA	13	11,126,421	12,109,287	1.75%	*THRB*
NBA	1	14,239,508	15,228,370	1.42%	*ESR1, CCDC170, ARMT1, RMND1, ZBTB2, AKAP12, MTHFD1L e ENSSSCG00000046251*
PV5	13	11,126,421	12,109,287	3.24%	*THRB*
PV5	2	135,494,632	136,455,843	0.98%	*FSTL4, C5orf15, VDAC1 e TCF7*
PV5	8	13,836,704	14,832,626	0.98%	*SLIT2*
PV5	4	10,836,287	11,818,923	0.97%	No gene found
PV5	1	255,680,248	256,676,846	0.95%	*PAPPA e ENSSSCG00000040472*

Abbreviations: NBA, number born alive; PV5, viable piglets at Day 5; SSC, *Sus scrofa chromosomes*; TNB, total number born.

Given the large number of genes identified in significant windows, we select only those windows explaining more than 0.90% of the genetic variance for further investigation, in a total of 4 windows for TNB, 2 for NBA, and 5 for PV5, which explained genetic variance greater than 0.95%, 1.42%, and 1.03%, respectively. Thus, only genes within these windows (Table 5) were prioritised for a deep analysis of their involvement with the development of the studied traits by a search in the literature. On the other hand, for the enrichment of BP and gene networks analyses, all genes present in the significant windows from GWAS were used (Supporting Information, Table S1).

Based on the functional annotation analysis for all genes identified in the significant windows of TNB, NBA, and PV5 (Supporting Information, Table S1), 35 genes were selected that are involved in BP that may explain the expression of these traits (Table 6).

**TABLE 6:** Genes in biological processes most related to the development of the three evaluated traits, identified in Ensembl BioMart.

Traits	SSC	Gene	Biological processes
TNB, NBA and PV5	1	*ZBTB2*	RNA polymerase II transcription, DNA methylation, immune system regulation, and cytokine production regulation
TNB, NBA and PV5	1	*MTHFD1L*	Neural tube closure, embryonic neurocranium and viscerocranium morphogenesis
TNB and NBA	1	*SHB*	Development of blood vessels, proliferation of B and T cells, proliferation of haematopoietic stem cells, and negative regulation of oocyte maturation
TNB	4	*OPRK1*	Defence response to viroses and immune response
TNB	4	*ST18*	Interleukin‐1 and interleukin‐6 mediated signalling, negative regulation of cell population proliferation
TNB and NBA	17	*KAT14*	Chromatin remodelling, involved in the regulation of embryonic development, cell division, and cell cycle
TNB and NBA	17	*POLR3F*	Positive regulation of innate immune response
TNB and NBA	17	*SLC24A3*	Transport and homeostasis of intracellular calcium ions and negative regulation of gene expression
TNB, NBA and PV5	8	*SLIT2*	Involved in the development of the nervous system
TNB	11	*FGF9*	Cellular differentiation, organ morphogenesis, regulation of cell division, fibroblast growth factor receptor signalling pathway, pulmonary development
TNB and PV5	14	*HMX3*	Cerebral development, embryo implantation, inner ear morphogenesis
TNB and NBA	16	*GLRA1*	Regulation of respiratory gas exchange, neuromuscular processes, nerve impulse transmission, muscle contraction, and startle response
TNB and NBA	16	*SPARC*	Cellular morphogenesis, angiogenesis, and migration of endothelial cells
TNB, NBA and PV5	13	*NKIRAS1*	Development of pulmonary alveoli, cellular response to estrogenic stimulus
TNB, NBA and PV5	1	*ESR1*	Cellular response to estrogenic stimulus
TNB, NBA and PV5	13	*THRB*	Molecular signals mediated by thyroid hormones and organ morphogenesis
TNB	4	*RB1CC1*	Innate immune response, liver, and heart development
TNB	8	*NKX3‐2*	Development of the pancreas and skeletal system
TNB and NBA	1	*PAX5*	Development of the cerebral cortex and differentiation of skeletal muscle cells
TNB and NBA	17	*OVOL2*	Formation of the endocardium, structures of the digestive tract, and embryonic vessels
NBA	9	*MR1*	Innate immune response and defence response to gram‐positive bacteria
NBA	9	*IER5*	Negative regulation of cell proliferation under stress, cellular response to thermal stress
NBA	18	*TAX1BP1*	Apoptosis
NBA	18	*HIBADH*	Catabolic process of valine
NBA	1	*THBS2*	Cell adhesion and negative regulation of angiogenesis
PV5	2	*FSTL4*	Cellular differentiation for specific structural characterisation of its function, negative regulation of brain‐derived neurotrophic factor receptor signalling pathway, and negative regulation of dendritic spine development
PV5	2	*TCF7*	Regulation of cellular activity by interleukin‐4 stimulation and inflammatory response
PV5	1	*SMOC2*	Positive regulation of angiogenesis and vascular wound healing, and migration of endothelial cells
PV5	4	*CCN1*	Cardiac morphogenesis, development of the ventricular septum, cell adhesion, and apoptotic process
PV5	8	*DHX15*	Innate immune defence response to bacteria and viruses
PV5	14	*EBF2*	Differentiation of brown adipose tissue cells, development of adipose tissue, and regulation of cold‐induced thermogenesis
PV5	14	*BNIP3L*	Cellular response to hypoxia, defence response to viruses, and regulation of programmed cell death
PV5	14	*ADRA1A*	Regulation of cardiac muscle contraction and vasoconstriction
PV5	13	*TGFBR2*	Vasculogenesis and embryonic development in the uterus
PV5	14	*DPYSL2*	Development of the nervous system and cellular differentiation

Abbreviations: NBA, number born alive; PV5, viable piglets at day 5; SSC, *Sus scrofa* chromosomes; TNB, total number born.

**FIGURE 4:**
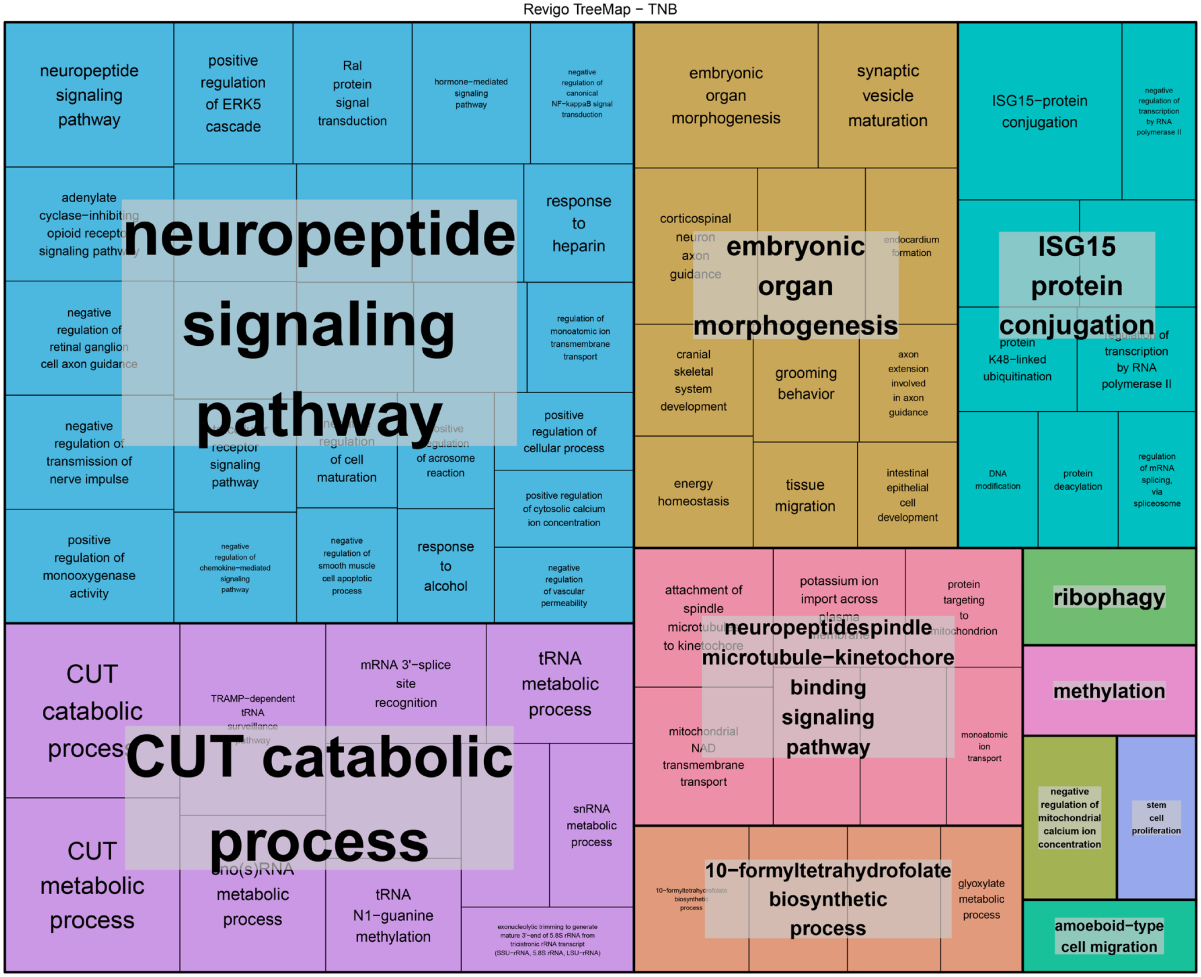
Superclusters of significant biological processes related to genes mapped in genomic windows associated with total number born (TNB). Enrichment analysis performed with PantherDB and summarised in REVIGO. [Colour figure can be viewed at wileyonlinelibrary.com]

**FIGURE 5:**
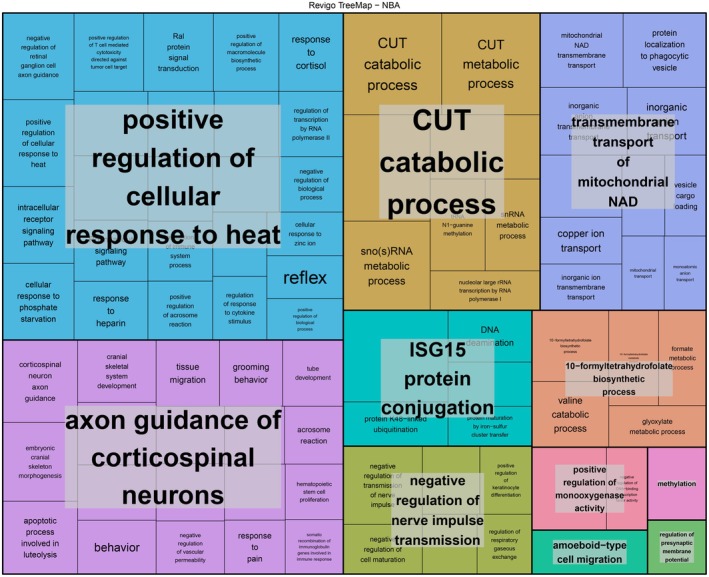
Superclusters of significant biological processes related to genes mapped in genomic windows associated with number born alive (NBA). Enrichment analysis performed with PantherDB and summarised in REVIGO. [Colour figure can be viewed at wileyonlinelibrary.com]

**FIGURE 6:**
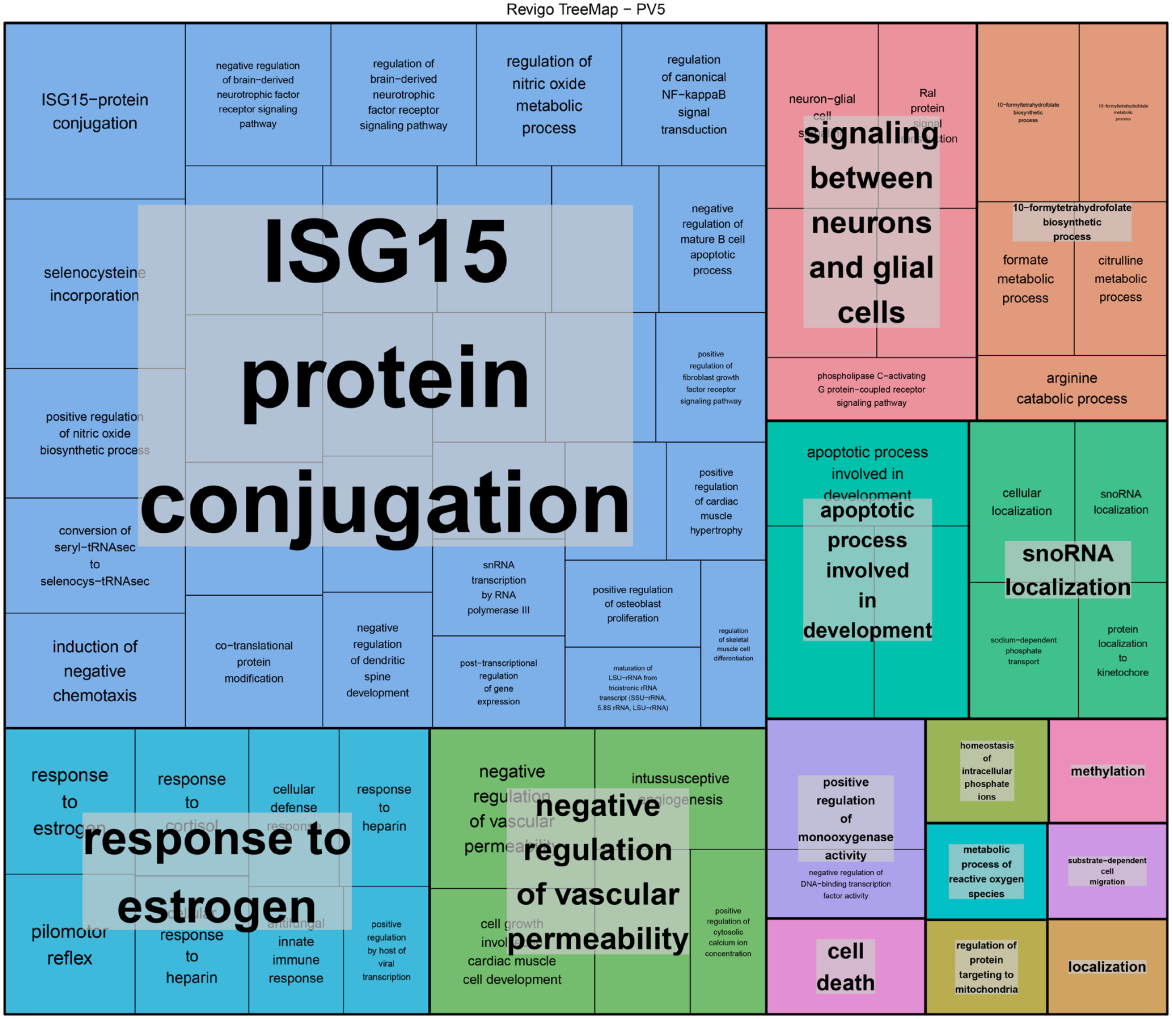
Superclusters of significant biological processes related to genes mapped in genomic windows associated with viable piglets at Day 5 (PV5). Enrichment analysis performed with PantherDB and summarised in REVIGO. [Colour figure can be viewed at wileyonlinelibrary.com]

Among the enriched BP identified with PANTHERdb and summarised with REVIGO in the superclusters (Figures 4, 5, 6), several showed high potential relevance to the target traits, such as embryonic organ morphogenesis, identified for TNB (Figure 4), which deserves to be highlighted. Another significant BP is the conjugation of the ISG15 protein, which may have an important role, since it was observed for all three traits analysed (Figures 4, 5, 6). Other BPs of interest include heat response regulation and nerve impulse transmission for NBA (Figure 5), as well as response to oestrogen and vascular permeability for PV5 (Figure 6).

**FIGURE 7:**
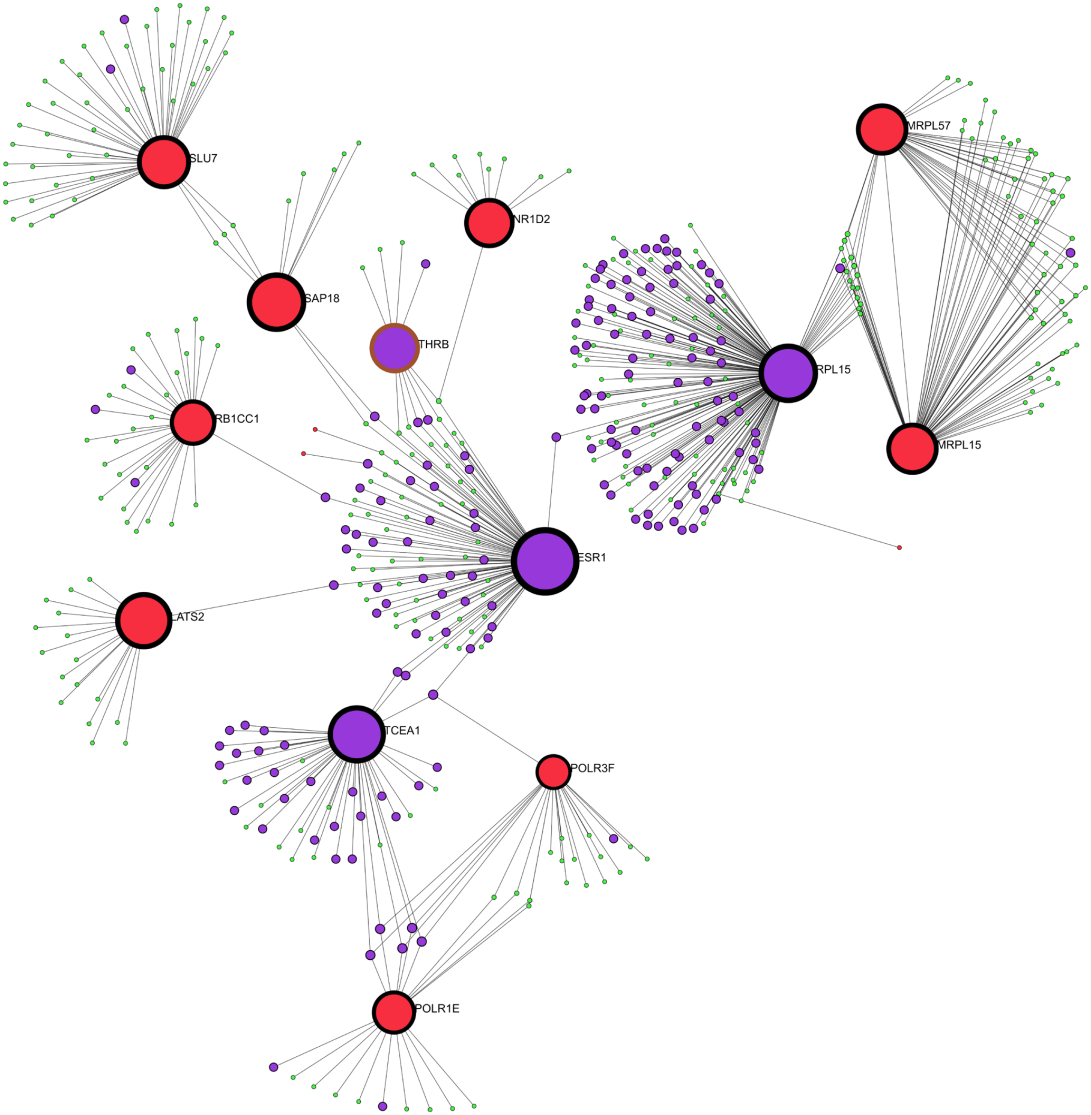
Gene interaction network constructed with genes located in the significant genomic windows identified in the GWAS associated with total number born (TNB), generated in Network Analyst using the STRING database with human‐derived data. Large circles represent GWAS‐identified genes for TNB. Purple circles denote genes/proteins associated with human reproductive processes. [Colour figure can be viewed at wileyonlinelibrary.com]

**FIGURE 8:**
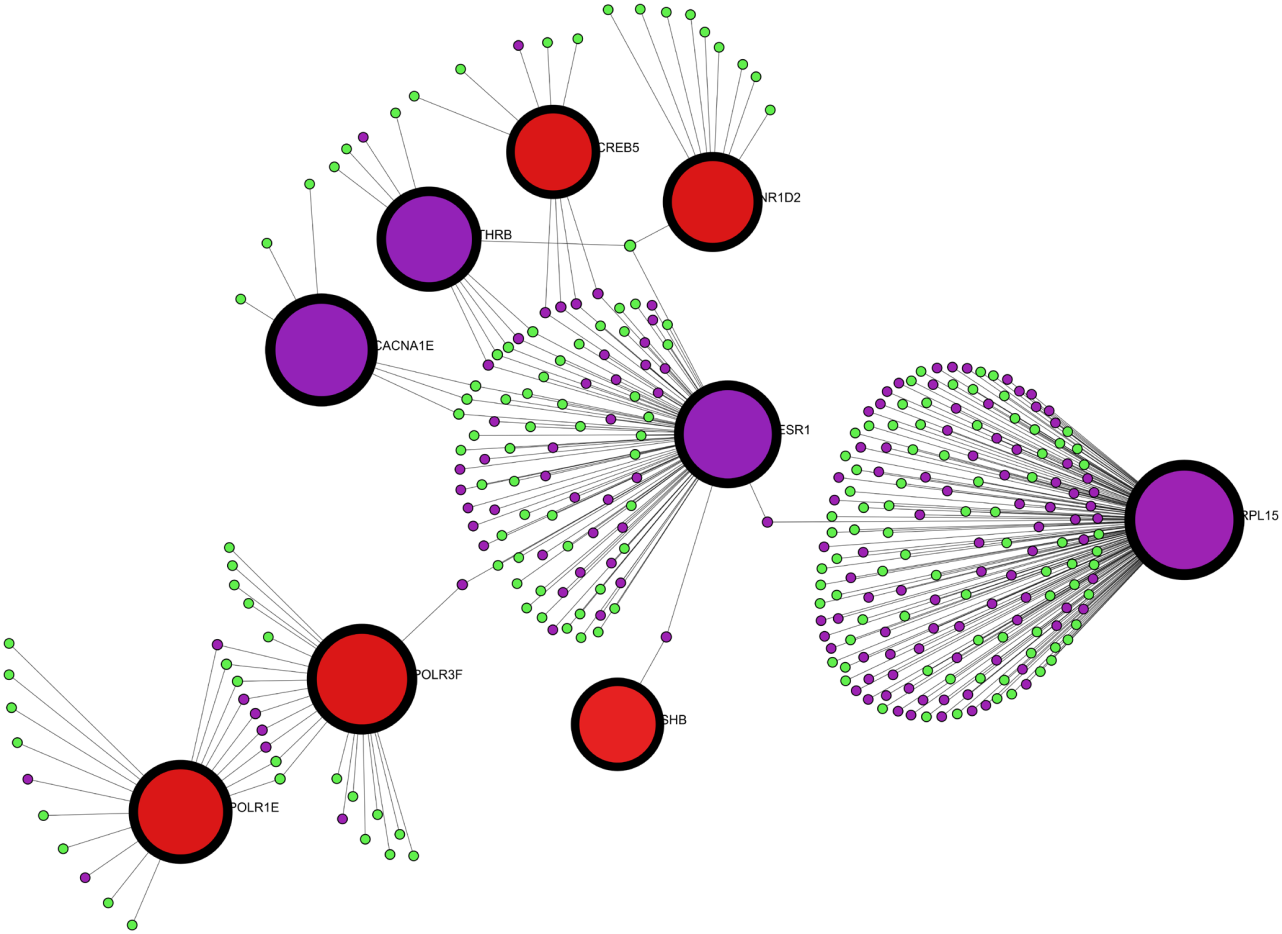
Gene interaction network constructed with genes located in the significant genomic windows identified in the GWAS associated with number born alive (NBA), generated in Network Analyst using the STRING database with human‐derived data. Large circles represent GWAS‐identified genes for NBA. Purple circles denote genes/proteins associated with human reproductive processes. [Colour figure can be viewed at wileyonlinelibrary.com]

**FIGURE 9:**
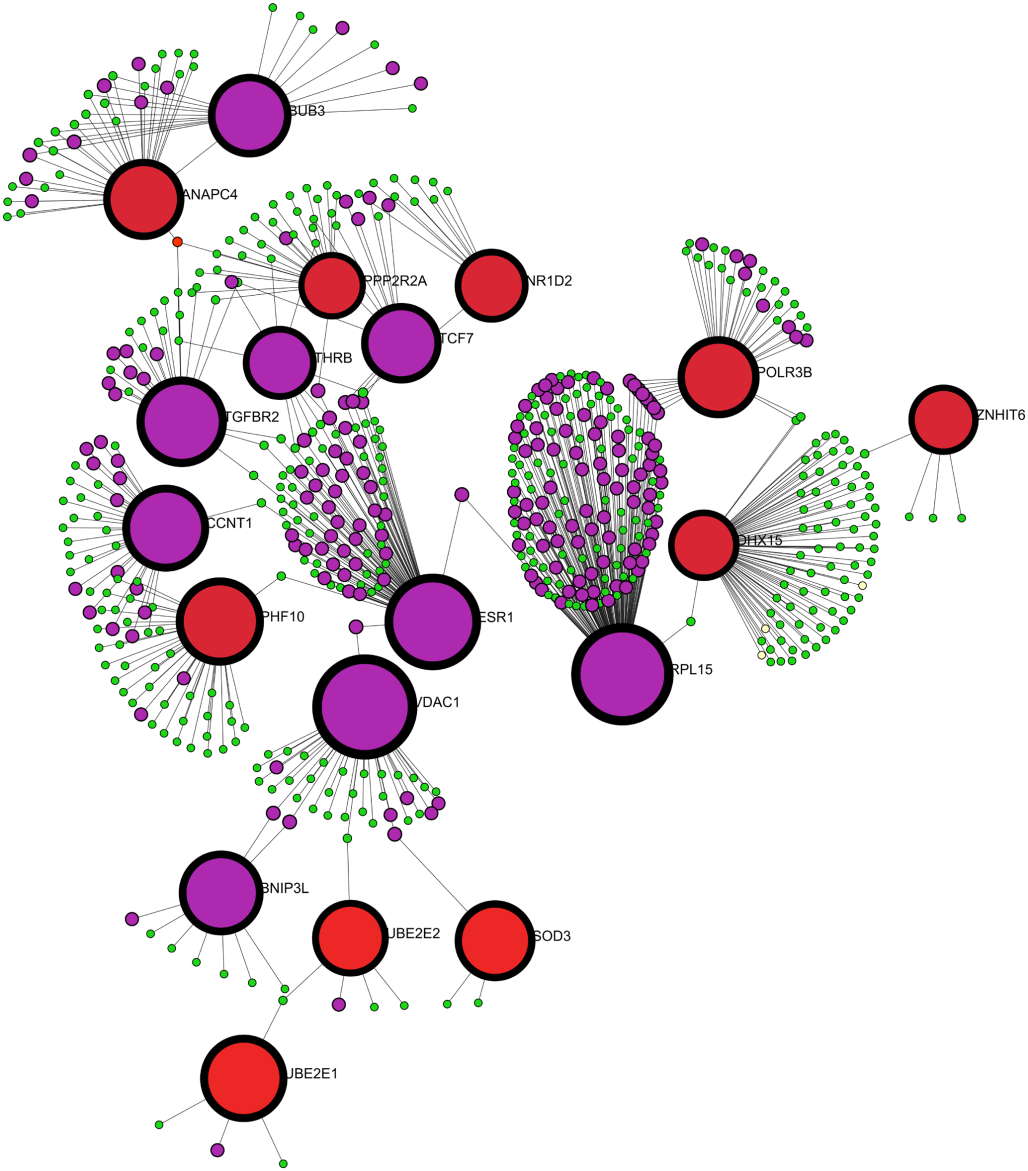
Gene interaction network constructed with genes located in the significant genomic windows identified in the GWAS associated with viable piglets at day 5 (PV5), generated in Network Analyst using the STRING database with human‐derived data. Large circles represent GWAS‐identified genes for PV5. Purple circles denote genes/proteins associated with human reproductive processes. [Colour figure can be viewed at wileyonlinelibrary.com]

The gene network construction was performed using all genes within significant windows for each trait. These networks revealed interactions among GWAS‐identified candidate genes and highlighted additional genes potentially indirectly associated with the development of TNB (Figure 7), NBA (Figure 8), and PV5 (Figure 9).

Given the primary interest in identifying genes linked to the productive capacity of females, the construction of gene networks enabled the identification of previously described genes associated with reproductive processes in humans. This information is relevant, as highlighted by Lunney et al. (2021), since swine models are increasingly used in human‐related research due to significant anatomical, physiological, and genomic similarities between the two species. Therefore, integrating GWAS with gene network analysis provides a powerful framework to elucidate the development of TNB, NBA, and PV5 in pigs.

## 4 Discussion

### 4.1 Heritability, Genetic and Phenotypic Correlations

In the present study, the *h* ^2^ estimates for TNB and NBA, derived from pedigree‐ and genomic‐based methods, were of low magnitude (Table 4), ranging from 0.12 ± 0.01 to 0.14 ± 0.01, showing similarities with results found in the literature for the same traits in the Large White breed. Arango et al. (2005), evaluating three different parities, obtained *h* ^2^ estimates ranging from 0.09 ± 0.04 to 0.11 ± 0.05 for TNB, and 0.09 ± 0.07 to 0.12 ± 0.10 for NBA. Zhang et al. (2019) and Lopez et al. (2017), using data from various farms in China and Korea, reported *h* ^2^ values for TNB and NBA ranging from 0.06 ± 0.00 to 0.12 ± 0.01. Conversely, Yang et al. (2023), also assessing different farms, reported *h* ^2^ estimates of 0.19 ± 0.00 for TNB and NBA, which, although still considered low, are considerably higher than those observed in our and other studies. Our results were also similar to those observed in other swine breeds and crosses, with ranges of 0.08 ± 0.03–0.19 ± 0.00 for TNB and 0.06 ± 0.00–0.19 ± 0.00 for NBA (Damgaard et al. 2003; Lopez et al. 2017; Zhang et al. 2019; Ogawa et al. 2020; Zaalberg et al. 2023; Yang et al. 2023).

Viable piglets at Day 5 is a complex trait whose genetic control is poorly understood. The *h* ^2^ estimates for PV5 obtained in the present study ranged from 0.10 ± 0.01 to 0.11 ± 0.01. To our knowledge, the first study addressing PV5 was conducted by Su et al. (2007), which analysed nucleus herd pigs from Danish Landrace and Yorkshire (Large White) populations, reporting *h* ^2^ of 0.09 ± 0.01 and 0.07 ± 0.01, respectively. Nielsen et al. (2013) evaluated populations pre‐selected for PV5 in the same breeds, obtaining uniform *h* ^2^ values (0.10 ± 0.01) for both breeds. Putz et al. (2015), working with Landrace and Large White pigs from a North Carolina genetics company, reported *h* ^2^ of 0.10 ± 0.02 (Landrace) and 0.11 ± 0.02 (Large White). In contrast, Su (2020), using three Danish lines (Duroc, Landrace, and Yorkshire) and two distinct models, found lower *h* ^2^ estimates than those previously mentioned, ranging from 0.01 to 0.04 for Duroc, 0.01 to 0.06 for Landrace, and 0.01 to 0.02 for Yorkshire. The result from our study corroborates most of the previous estimates, except those from Su (2020). This discrepancy may be due to the intensive selection criteria for PV5 applied in Danish populations, which could have reduced the additive genetic variance and, consequently, the *h* ^2^ estimates. In contrast, in other populations, including ours, the trait PV5 has been selected with less intensity, possibly resulting in a slightly higher *h* ^2^.

The low *h* ^2^estimates obtained for the three traits indicate that they are strongly influenced by environmental and management factors (Guo et al. 2016; Jiang et al. 2019). Low *h* ^2^ estimates demonstrate that little of the trait variation can be explained by breeding values, indicating a weaker relationship between parental genotypes and phenotypic expression in offspring (Sell‐Kubiak et al. 2022). Although low *h* ^2^ traits are more challenging for selection, they can still be used to improve herds; however, they tend to exhibit slow fixation across generations (Sell‐Kubiak 2021).

Despite the low *h* ^2^ observed with both methodologies, the genetic and phenotypic correlations among TNB, NBA and PV5 were high and positive (Table 4), enabling their effective use in selection. The genetic correlation between TNB and NBA was 0.94 ± 0.01. Similar results for these traits were reported by Roehe and Kennedy (1995) and Zhang et al. (2019) in populations of Large White, Landrace, and Duroc breeds, with correlations ranging from 0.94 ± 0.00 to 0.99 ± 0.01. Therefore, selecting for either TNB or NBA is expected to have a strongly correlated response in the same direction for the other trait.

The *r*_*g*_ and *r*_*p*_ correlations between PV5 and the other two traits were also high and positive (Table 4), but lower than those observed between TNB and NBA, indicating reduced efficiency in selection response when PV5 is not prioritised. As for the *h* ^2^ estimates, few studies report correlation for PV5, and the existing studies show discrepancies among them. Su et al. (2007) reported moderate *r*_*g*_ (0.34 ± 0.11) between TNB and PV5 in Landrace, contrasting with higher *r*_*g*_ values of 0.74 ± 0.03 found by Nielsen et al. (2013) and 0.85 ± 0.02 by Putz et al. (2015) for the same breed, which corroborate with our results (0.74 ± 0.05 and 0.76 ± 0.04 via pedigree and genomic methods, respectively). For Yorkshire (Large White), Su et al. (2007) reported *r*_*g*_ and *r*_*p*_ of 0.57 ± 0.12 and 0.64 ± 0.01, lower than our estimates but still considered high and positive, whereas Nielsen et al. (2013) and Putz et al. (2015) observed *r*_*g*_ values of 0.77 ± 0.00 and 0.73 ± 0.01. Regarding the PV5‐NBA relationship, only Su et al. (2007) provided *r*_*g*_ estimates of 0.83 ± 0.00 (Landrace) and 0.81 ± 0.00 (Yorkshire), like those obtained in our study. Discrepancies in PV5 correlations across the available studies may be due to the differences in the datasets used. Su et al. (2007), pioneers in estimating genetic parameters for PV5, used data from herds selected for TNB, observing that the increase in TNB resulted in a high perinatal mortality, potentially explaining their lower correlation between TNB and PV5. In contrast, Nielsen et al. (2013) analysed herds pre‐selected for PV5, likely exhibiting stabilised rates for both TNB and PV5.

Our results, combined with those from the literature, demonstrate that *h* ^2^ for these and other reproductive traits is of low magnitude. Although selection response for these traits is slow, the strong genetic correlation among traits indicates that selecting for one trait indirectly influences the others, yielding positive responses concurrently. This effect was demonstrated by Hsu and Johnson (2014), who evidenced that highly correlated traits, whether positively or negatively, tend to respond to selection in the same direction after 28 generations of swine selection.

### 4.2 Positional and Functional Candidate Genes for TNB, NBA and PV5

Litter traits are critical productivity indicators in swine nucleus herds. However, as observed in our study, reproductive traits are influenced by multiple genomic regions distributed across various chromosomes (Figures 1, 2, 3). For each significant window identified, a substantial number of positional candidate genes were observed (Supporting Information, Table S1), highlighting a complex genomic architecture shaped by thousands of genes with minor effects that collectively regulate the target traits (Guo et al. 2016; Ding et al. 2021; Zhao et al. 2022). Identifying and characterising the full set of genes within significant windows (Supporting Information, Table S1) is essential to advance our understanding of the genetic architecture of complex traits, particularly those with low *h* ^2^. Nevertheless, the following discussion will focus solely on positional candidate genes located in windows explaining the highest genetic variance (> 0.90%) for at least one of the evaluated traits, taking into account their biological functions, BP, and the gene network. If those genes were also located in significant windows for other litter traits, they were also discussed, giving emphasis to the novel findings.

In our study, the genes associated with TNB, NBA and PV5 were enriched in multiple BPs (Supporting Information, Tables S2–S4), and it is possible to highlight those related to the morphogenesis pathway for TNB (Figure 4) and neurogenesis for NBA (Figure 5). Genes linked to embryogenesis are essential, as endogenous or exogenous changes during fetal development directly influence embryonic success (Biswas et al. 2018). Positional candidate genes, such as *SLIT2* and *MTHFD1L*, related to neural tube integrity and nephrogenesis, as well as *OVOL2*, involved in cardiac and intestinal development, are also important functional candidates for the genetic control of the evaluated traits. In addition, BPs related to environmental stressors and heat response processes were also enriched for NBA (Figure 5) and may relate to thermal stress adaptation, a significant challenge in pig production. Heat‐stressed animals exhibit reduced feed intake and seasonal infertility (Ross et al. 2017). Intrauterine heat stress during gestation leads to postnatal immune challenges and lower piglet birth weights (Johnson et al. 2020), increasing postnatal mortality.

The oestrogen response BP, enriched for PV5 (Figure 6), is essential for piglet viability, regulating reproductive, skeletal, and cardiovascular development (Paterni et al. 2014). Furthermore, catabolic processes related to cryptic unstable transcripts (CUTs) were enriched for TNB and NBA, suggesting its function on gene expression control, essential for cellular regulation during embryogenesis. Evidences link CUTs to glucose catabolism and transcriptional regulation (Neil et al. 2009). Increased cryptic transcription factor expression was associated with stem cell aging in mammals (Mccauley and Dang 2021). Therefore, the association of CUT with glucose homeostasis and aging stem cells might affect cellular integrity. If dysregulation occurs in those processes at the embryonic level, it can harm the maintenance of pregnancy; consequently, affecting the traits evaluated in our study. Moreover, the ISG15 protein conjugation supercluster became evident in the three litter traits (Figures 4, 5, 6), demonstrating its crucial role in preserving piglet genomic integrity and female fertility. ISG15 has been implicated in several cellular activities, and *ISG15* knockout in mice improves reproductive capacity by enhancing the ovulation rate (Chen et al. 2023). Thus, the identified BP superclusters indicate that the positional candidate genes found in our study are also functional for the evaluated litter traits.

#### 4.2.1 Genes Involved With Reproductive Hormone Processes

The oestrogen receptor 1 (*ESR1*) gene, located in the second‐highest variance window (Table 5) for TNB and NBA, and identified in the gene network of all traits (Figures 7, 8, 9), is mapped to SSC1 and encodes oestrogen receptors (Muñoz et al. 2007). The identification of the *ESR1* gene in the present study serves as a robust biological validation of our GWAS results, given its historically consolidated role in regulating reproductive physiology and litter size in swine (Rothchild et al. 1996; Muñoz et al. 2007; Cieleń and Sell‐Kubiak 2024). Nevertheless, one of the novel contributions of our study lies in expanding the understanding of the *ESR1* functional landscape. Our gene network analysis demonstrates its interaction with diverse positional candidate genes that may also be involved in the regulation of the studied traits, including *THRB* (thyroid hormone receptor beta), *TCEA1* (transcription elongation factor A1), *VDAC1* (voltage dependent anion channel 1), *RPL15* (ribosomal protein L15), and *CACNA1* (calcium voltage‐gated channel subunit alpha1) genes. Collectively, these findings suggest that reproductive efficiency is modulated by a complex crosstalk between established oestrogen signalling and other biological pathways represented by these novel candidates.

The thyroid hormone receptor beta (*THRB*) gene was identified within the highest‐significance genomic window for all three traits on SSC13 and is involved in the BP of thyroid hormone response and morphogenesis (Table 6). In addition, *THRB* was highlighted in the gene network analyses as being associated with reproductive processes in humans (Figures 7, 8, 9). This gene encodes isoforms of the thyroid hormone receptor, a class of hormones essential for regulating metabolism, as well as the proliferation and differentiation of bone cells in mice (Kim and Mohan 2013). Moreover, *THRB* influences the metabolism and development of ovarian, uterine, and placental tissues (Silva et al. 2018). In swine, this gene has also been associated with metabolic disorders and postnatal developmental failures (Loueva et al. 2000). Therefore, our results extend the previously reported association of *THRB* with TNB (Wu et al. 2022) by revealing a novel association with PV5, which explained 3.24% of its additive genetic variance, suggesting a pleiotropic effect of this gene on both sow fertility and offspring viability. Given its role in metabolism and development, mutations in *THRB* may potentially compromise piglet natality and survival.

#### 4.2.2 Genes Involved With Embryonic Development

The slit guidance ligand 2 (*SLIT2*) gene, associated with all three traits (Supporting Information, Table S1) and located in the second‐highest variance window (Table 5) for PV5 (SSC8), acts in nervous system development (Table 6). Induced deletion of *SLIT2* in mice disrupted nephrogenesis and caused glomerular vascularization defects (Li et al. 2021). This gene was already reported in gene expression comparisons of Meishan and Duroc swine endometrium as upregulated in Meishan (Zhang, Liu, et al. 2023; Zhang, Yao, et al. 2023), a breed renowned for its hyperprolificity (Zhou et al. 2023). In addition, *SLIT2* has been related to epithelial cell differentiation, fertility regulation, and ovulation BP (Zhang, Liu, et al. 2023; Zhang, Yao, et al. 2023). Although this gene had been previously related to swine fertility, our study is the first to report an association of *SLIT2* with TNB, NBA, and PV5. Thus, our findings suggest that mutations in this gene may affect female fertility and fetal tissue development, thereby directly impacting piglet natality and postnatal viability.

The ovo like zinc finger 2 (*OVOL2*) gene, associated with TNB and NBA in our study, is located on the third‐highest variance window for TNB in SSC17 (Table 5). This gene is involved in cardiac and digestive cell differentiation during embryogenesis (Table 6). Although it has not been previously associated with reproductive traits in pigs, *OVOL2* is critical for neurocranial development and cardiac/intestinal cell differentiation in mice (Mackay et al. 2006; Unezaki et al. 2007). Mutations in *OVOL2* may impair embryonic development, leading to lethality and directly impacting TNB and NBA. In addition, such mutations may contribute to fetal malformations and congenital disorders which, due to their high correlation with PV5, could negatively influence piglet survival after birth.

The sh2 domain containing adaptor protein B (*SHB*) gene, located in a high‐variance window for TNB (Table 5) on SSC1, and among significant windows for NBA (Supporting Information, Table S1), regulates haematopoietic cell proliferation and oocyte maturation (Table 6). Though not directly linked to human reproductive processes in the NBA gene network (Figure 8), *SHB* knockout mice exhibited embryonic malformations (Kriz et al. 2007) and ovarian follicle maturation defects (Calounova et al. 2010). This gene has also been associated with reduced haematopoietic stem cells (Gustafsson et al. 2013). The *SHB* gene was not previously associated with litter traits in pigs. However, its role in embryonic development suggests an important contribution to fertility and, consequently, to birth‐related traits. Therefore, mutations in the *SHB* gene may impair the development of reproductive cells, as well as embryonic development, thereby reducing the number of viable embryos and live‐born piglets.

The methylenetetrahydrofolate dehydrogenase 1‐like (*MTHFD1L*) gene, associated with TNB and NBA, is located on SSC1 within the second‐highest variance genomic window (Table 5), and is involved with neural tube formation and embryonic neurocranial development (Table 6). In rats, *MTHFD1L* deletion reduced cranial mesenchymal density (Shin et al. 2019), impairing embryogenesis (Momb et al. 2012). Although this gene has previously been associated with carcass traits (Liu et al. 2023), it has not been linked to piglet‐related traits. Hence, mutations in *MTHFD1L* may lead to neurocranial malformations, negatively affecting embryogenesis and potentially increasing stillbirth rates.

The exocyst complex component 3 like 2 (*EXOSC3*) gene, associated with TNB and NBA, is located in the fourth‐highest variance window for TNB (Table 5) on SSC1. This gene has not been previously reported as associated with fertility or birth‐related traits in either animals or humans. However, *EXOSC3* mutations are involved with pontocerebellar hypoplasia in humans, characterised by brain atrophy (Halevy et al. 2014; François‐Moutal et al. 2018). Given the physiological similarities between swine and humans (Lunney et al. 2021), mutations in this gene may impair neurogenesis or neurological development, potentially increasing stillbirths.

#### 4.2.3 Genes Involved With Growth Factors

The follistatin like 4 (*FSTL4*) gene, encoding the follistatin‐related protein 4, is located on SSC2 and was identified in the second‐highest significance window for PV5 (Table 5). Although this gene has previously been associated with swine body size (Banestani et al. 2023), embryonic development (Zhang, Liu, et al. 2023; Zhang, Yao, et al. 2023), and follicle‐stimulating hormone (FSH) expression (Lu et al. 2021), this is the first time that *FSTL4* is associated with piglet viability. Given that the viability of newborn piglets is directly influenced by body mass gain and growth during the first days of life (Baxter et al. 2008), mutations in the *FSTL4* gene may affect both embryonic and postnatal development, leading to a reduced number of viable piglets. The pappalysin 1 (*PAPPA*) gene was identified in the fifth‐highest variance window for PV5 (Table 5) and mapped to SSC1. This gene has previously been described as a candidate for growth factors, meat quality (Green et al. 2023), body conformation, and digit disorders (Fan et al. 2009) in pigs. However, this is the first report associating *PAPPA* with PV5, suggesting that mutations in this gene may affect body development, contributing to morphological anomalies, potentially compromising piglet viability.

#### 4.2.4 Genes Involved With Immunity

The Transcription Factor 7 (*TCF7*) gene, identified in the second‐highest variance window for PV5 (Table 5), is located on SSC2 and it is involved in BP linked to interleukin‐mediated inflammatory factors (Table 6). This gene was also detected in the gene network (Figure 8) and has known roles in human reproductive processes. In swine, *TCF7* was already related to increased susceptibility to respiratory diseases (Xu et al. 2019). In addition, sows experiencing respiratory infections during gestation can increase their piglets' vulnerability to bacterial challenges (Feng et al. 2001). Although *TCF7* has been linked to health traits in pigs, this is the first report of its association with piglet survival, specifically with PV5. These results suggest that mutations in this gene may predispose inflammatory responses in gestating sows, impairing the immune response of their offspring and, consequently, increasing their susceptibility to diseases that could lead to mortality.

The zinc finger and BTB domain containing 2 (*ZBTB2*) gene, identified in the genomic window explaining the second highest genetic variance for TNB and NBA on SSC 1 (Table 5), was also significant for PV5 (Supporting Information, Table S1). This gene has not previously been associated with NBA or PV5, although it has been proposed as a candidate for TNB (Martins et al. 2022). The *ZBTB2* is involved in BP related to cytokine modulation and production (Table 6). Cytokines regulate immune function, and their dysregulation can lead to heightened inflammation or immunosuppression (Barten et al. 2006). Moreover, *ZBTB2* showed differential expression in the endometrium during early gestation stages in swine, suggesting a critical role in embryo implantation (Pierzchała et al. 2020). Thus, mutations in this gene may affect embryo implantation and immune responses, potentially leading to embryonic mortality or increased disease susceptibility, which could negatively impact piglet survival.

#### 4.2.5 Genes Involved With Regulation and Cellular Homeostasis

The solute carrier family 24 member 3 (*SLC24A3*) gene, identified in the third‐highest genetic variance window for TNB (Table 5) on SSC 17, was also significantly associated with NBA (Supporting Information, Table S1). This gene has previously been associated with birth and reproductive traits in swine (Ma et al. 2019; Lian et al. 2022), supporting the results of our study. In mice, uterine expression of *SLC24A3* increased during the estrous cycle. Moreover, *SLC24A3* is shown to interact with proteins mediating calcium and potassium transport across cell membranes, suggesting potential regulatory roles in reproductive functions (Yang et al. 2010). Furthermore, *SLC24A3* is involved in BP related to intracellular calcium ion transport, calcium homeostasis, and negative regulation of gene expression (Table 6). In hyperprolific sows, elevated calcium ion concentrations were observed (Ma et al. 2019), which may indicate a critical role for *SLC24A3* in embryonic survival. Therefore, this gene may be considered an important candidate for TNB and NBA, as it could be involved in reproductive functions, including embryo maintenance.

The voltage dependent anion channel 1 (*VDAC1*) gene, located on SSC2, was another new candidate identified in the second‐highest significance window for PV5 (Table 5). This gene was also highlighted in the gene ontology analysis (Supporting Information, Table S4) and encodes a mitochondrial transmembrane ion channel protein that is critical for ATP production (Osellame 2012). In humans, *VDAC1* expression increases in follicular fluid under high estradiol levels, correlating positively with viable embryo counts (Mazloomi et al. 2022). In swine, *VDAC1* expression rises significantly during postnatal epididymal development in males (Fang et al. 2023), and in females it has been localised across the oocyte surface (Cassara et al. 2010). Studies suggest that *VDAC1* regulates mitochondrial remodelling and mitophagy during oocyte development in Drosophila (Ng et al. 2024). Mitochondrial integrity is essential for oocyte maturation and embryo viability (Ashrafi and Schwarz 2013; Boucret et al. 2015). Thus, *VDAC1* is a strong candidate for reproductive traits and, consequently, may influence piglet survival, as it is involved in ATP production, the primary energy molecule in cellular metabolism, which is essential for survival.

In addition to the previously described genes, some uncharacterized candidates such as ENSSSCG00000058091 and ENSSSCG00000040472 have never been associated with reproductive traits in livestock. Nonetheless, in our study, these genes were associated, respectively, with TNB and PV5 in the highest genetic variance windows of SSC1 (Table 5). ENSSSCG00000058091 is involved with intracellular mitochondrial homeostasis, which is essential for ATP production (Supporting Information, Table S2), while ENSSSCG00000040472 acts on cytoplasmic protein synthesis (Ensembl BioMart), which are BP of great relevance for TNB and PV5. Therefore, these novel findings are important for supporting the elucidation of the functions of these genes, including their roles in the evaluated traits.

In this study, we used a large number of animals from a nucleus farm, and a robust methodology for genetic parameter estimates and to perform GWAS for TNB, NBA, and PV5. The windows variance‐based GWAS approach is helpful to find genomic regions involved with polygenic traits, although some methodological limitations exist. The aggregation of multiple SNPs in fixed 1 Mb windows limits the identification of individual causal variants. However, for reproductive traits, which are generally polygenic, the existence of a single SNP with a large effect is unlikely, which reduces the probability of failing to detect a causal SNP due to the use of windows. Another limitation is the LD variation across the genome, since windows larger than the actual LD blocks may include recombination regions. Nevertheless, LD analysis in the studied population showed high mean *r* ^2^ values for 1 Mb windows across all chromosomes (Supporting Information, Figure S1), indicating that these regions adequately represent the inherited haplotype blocks. Although windows variance‐based GWAS does not provide direct measures of statistical significance, a strict threshold was applied, selecting regions that explain more than 10 times the expected genetic variance for each trait, which is expected to reduce the probability of false‐positive associations. Moreover, the positional candidate genes found in our study were involved in critical BP for the development of such traits, which reinforces the reliability of the results. Hence, our findings contribute to expanding the knowledge on the genetic mechanisms underlying TNB, NBA, and PV5. However, further functional validation is critical to confirm the roles of new findings in the expression of the evaluated traits. Furthermore, our results may help develop breeding strategies aiming to optimise reproductive efficiency. Nevertheless, validation is crucial to ensure that those associations are maintained before their use in other populations.

## 5 Conclusions

The reproductive traits TNB, NBA and PV5 exhibit low heritability; however, the strong genetic and phenotypic correlation between them allows indirect selection gains. Analysis of genes located in high‐variance windows and identified in the interaction networks are promising genetic markers. Key positional candidates include *ESR1, THRB, ZBTB2*, and *SLC24A3*, which are functionally linked to the studied traits. Novel associations with TNB, NBA or PV5 were found for *MTHFD1L*, *SHB, SLIT2, FSTL4, PAPPA, VDAC1, TCF7* and *OVOL2*, which influence similar traits in other species. The ENSSSCG00000058091 and ENSSSCG00000040472 genes and the newly implicated *EXOSC3* were also identified as potentially associated with litter traits. All these genes are involved in biological processes essential to the development of TNB, NBA and PV5. Therefore, our findings contribute to a better understanding of the genetic architecture of TNB, NBA and PV5, which have great impact on swine production efficiency. Moreover, the positional and functional candidate genes identified in our study are potential markers to be used in selection to improve litter traits after appropriate validation.

## Funding

This research received financial support from the Brazilian Agricultural Research Corporation (Embrapa), Brazilian Government, project #20.23.08.005.00.00.

## Ethics Statement

All methods and procedures used in this study were reviewed and approved by the Ethics Committee on Animal Use (CEUA) from Embrapa Swine and Poultry National Research Center, under protocol #002/2016, in agreement with the rules established by the National Council of Animal Experimentation Control (CONCEA) to ensure compliance with international guidelines for animal welfare.

## Conflicts of Interest

The authors declare no conflicts of interest.

## Supporting information


**Data S1:** jbg70042‐sup‐0001‐Supinfo.xlsx.

## Data Availability

The data that support the findings of our study are provided within the manuscript and its Supporting Information. Other datasets analysed in this study require permission from the BRF S.A. company for their use, which can be requested through the corresponding author.

## References

[jbg70042-bib-0001] Aguilar, I. , I. Misztal , D. L. Johnson , A. Legarra , S. Tsuruta , and T. J. Lawlor . 2010. “Hot Topic: A Unified Approach to Utilize Phenotypic, Full Pedigree, and Genomic Information for Genetic Evaluation of Holstein Final Score.” Journal of Dairy Science 93, no. 2: 743–752. 10.3168/jds.2009-2730. 20105546

[jbg70042-bib-0002] Aguilar, I. , I. Misztal , S. Tsuruta , et al. 2014. “PREGSF90‐POSTGSF90: Computational Tools for the Implementation of Single‐Step Genomic Selection and Genome‐Wide Association With Ungenotyped Individuals in BLUPF90 Programs Andres Legarra Council on Dairy Cattle Breeding. 10. World Congress on Genetics Applied to Livestock Production (WCGALP). American Society of Animal Science.” 10.13140/2.1.4801.5045.

[jbg70042-bib-0003] Alves, K. 2018. “Estimation of Direct and Maternal Genetic Parameters for Individual Birth Weight, Weaning Weight, and Probe Weight in Yorkshire and Landrace Pigs.” Journal of Animal Science 96, no. 7: 2567–2578. 10.1093/jas/sky172. 29762734 PMC6095450

[jbg70042-bib-0004] Amberger, J. S. , C. A. Bocchini , A. F. Scott , and A. Hamosh . 2018. “OMIM.Org; Leveraging Knowledge Across Phenotype‐Gene Relationships.” Nucleic Acids Research 47, no. D1: D1038–D1043. 10.1093/nar/gky1151. PMC6323937 30445645

[jbg70042-bib-0094] Arango, J. , I. Misztal , S. Tsuruta , M. Culbertson , and W. Herring . 2005. “Threshold‐Linear Estimation of Genetic Parameters for Farrowing Mortality, Litter Size, and Test Performance of Large White Sows.” Journal of Animal Science 83, no. 3: 499–506. 10.2527/2005.833499x. 15705745

[jbg70042-bib-0005] Ashrafi, G. , and T. L. Schwarz . 2013. “The Pathways of Mitophagy for Quality Control and Clearance of Mitochondria.” Cell Death and Differentiation 20: 31–42. 10.1038/cdd.2012.81. 22743996 PMC3524633

[jbg70042-bib-0006] Banestani, E. S. , E. Sanjari Banestani , A. Esmailizadeh , M. Momen , A. Ayatollahi Mehrgardi , and M. Mokhtari . 2023. “Genome‐Wide Association Study Identifies Significant SNP and Related Genes Associated With Body Size in Yorkshire Pigs Using Latent Variable Modelling.” Journal of Agricultural Science 161, no. 4: 599–605. 10.1017/S0021859623000424.

[jbg70042-bib-0007] Barten, M. J. , A. Rahmel , J. Bocsi , et al. 2006. “Cytokine Analysis to Predict Immunosuppression.” Cytokine 33, no. 6: 374–378. 10.1002/cyto.a.20215. 16479614

[jbg70042-bib-0008] Baxter, E. M. , S. Jarvis , R. B. D'Eath , et al. 2008. “Investigating the Behavioural and Physiological Indicators of Neonatal Survival in Pigs.” Theriogenology 69, no. 6: 773–783. 10.1016/j.theriogenology.2007.12.007. 18242685

[jbg70042-bib-0009] Biswas, D. , K. H. So , S. U. Hwang , et al. 2018. “Embryotropic Effects of Vascular Endothelial Growth Factor on Porcine Embryos Produced by In Vitro Fertilization.” Theriogenology 120: 147–156. 10.1016/j.theriogenology.2018.07.024. 30121547

[jbg70042-bib-0010] Boucret, L. , J. M. Chao Barca , C. Morinière , et al. 2015. “Relationship Between Diminished Ovarian Reserve and Mitochondrial Biogenesis in Cumulus Cells.” Human Reproduction 30, no. 7: 1653–1664. 10.1093/humrep/dev114. 25994667

[jbg70042-bib-0011] Calounova, G. , G. Livera , X. Zhang , et al. 2010. “The Src Homology 2 Domain‐Containing Adapter Protein B (SHB) Regulates Mouse Oocyte Maturation.” PLoS One 5, no. 6: e11155. 10.1371/journal.pone.0011155. 20585392 PMC2886836

[jbg70042-bib-0012] Cassara, M. C. , M. . C. Cassará , V. . A. Menzel , K.‐D. Hinsch , C. Wrenzycki , and E. Hinsch . 2010. “Voltage‐Dependent Anion Channels 1 and 2 Are Expressed in Porcine Oocytes.” Bioscience Reports 30, no. 3: 193–200. 10.1042/BSR20090088. 19630752

[jbg70042-bib-0013] Chen, Y. , J. Zhou , S. Wu , et al. 2023. “ISG15 Suppresses Ovulation and Female Fertility by ISGylating ADAMTS1.” Cell & Bioscience 13, no. 84: 4. 10.1186/s13578-023-01024-4. 37170317 PMC10176748

[jbg70042-bib-0014] Cheng, J. , R. Fernando , H. Cheng , et al. 2021. “Genome‐Wide Association Study of Disease Resilience Traits From a Natural Polymicrobial Disease Challenge Model in Pigs Identifies the Importance of the Major Histocompatibility Complex Region.” G3 (Bethesda) 28, no. 12: jkab441. 10.1093/g3journal/jkab441. PMC9210302 35100362

[jbg70042-bib-0015] Cieleń, G. , and E. Sell‐Kubiak . 2024. “Importance and Variability of the Paternal Component in Sow Reproductive Traits.” Animal Genetics 65: 853–866. 10.1007/s13353-024-00910-y. PMC11561000 39422876

[jbg70042-bib-0016] Cleveland, M. A. , and J. M. Hickey . 2013. “Practical Implementation of Cost‐Effective Genomic Selection in Commercial Pig Breeding Using Imputation.” Journal of Animal Science 91, no. 8: 3583–3592. 10.2527/jas.2013-6270. 23736050

[jbg70042-bib-0017] Damgaard, L. H. , L. Rydhmer , P. Løvendahl , et al. 2003. “Genetic Parameters for Within‐Litter Variation in Piglet Birth Weight and Change in Within‐Litter Variation During Suckling.” Journal of Animal Science 81, no. 3: 604–610. 10.2527/2003.813604x. 12661639

[jbg70042-bib-0018] Dekkers, J. C. M. 2012. “Application of Genomics Tools to Animal Breeding.” Current Genomics 13, no. 3: 207–212. 10.2174/138920212800543057. 23115522 PMC3382275

[jbg70042-bib-0019] Ding, R. , Y. Qiu , Z. Zhuang , et al. 2021. “Genome‐Wide Association Studies Reveal Polygenic Genetic Architecture of Litter Traits in Duroc Pigs.” Theriogenology 173: 269–278. 10.1016/j.theriogenology.2021.08.012. 34403972

[jbg70042-bib-0020] Fan, B. , S. K. Onteru , B. E. Mote , T. Serenius , K. J. Stalder , and M. F. Rothschild . 2009. “Large‐Scale Association Study for Structural Soundness and Leg Locomotion Traits in the Pig.” Genetics Selection Evolution 41: 14. 10.1186/1297-9686-41-14. PMC2657774 19284518

[jbg70042-bib-0021] Fang, S. , Z. Li , S. Pang , Y. Gan , X. Ding , and H. Peng . 2023. “Identification of Postnatal Development Dependent Genes and Proteins in Porcine Epididymis.” BMC Genomics 24: 729. 10.1186/s12864-023-09827-y. 38049726 PMC10694963

[jbg70042-bib-0022] Feng, W. , W.‐h. Feng , S. M. Laster , et al. 2001. “In Utero Infection by Porcine Reproductive and Respiratory Syndrome Virus Is Sufficient to Increase Susceptibility of Piglets to Challenge by *Streptococcus suis* Type II.” Journal of Virology 75, no. 10: 4889–4895. 10.1128/JVI.75.10.4889-4895.2001. 11312360 PMC114243

[jbg70042-bib-0096] François‐Moutal, L. , S. Jahanbakhsh , A. D. L. Nelson , et al. 2018. “A Chemical Biology Approach to Model Pontocerebellar Hypoplasia Type 1b (PCH1B).” ACS Chemical Biology 13, no. 10: 3000–3010. 10.1021/acschembio.8b00745. 30141626 PMC6504997

[jbg70042-bib-0023] Green, H. E. , H. R. de Oliveira , A. B. Alvarenga , et al. 2023. “Genomic Background of Biotypes Related to Growth, Carcass and Meat Quality Traits in Duroc Pigs Based on Principal Component Analysis.” Journal of Animal Breeding and Genetics 141: 12831. 10.1111/jbg.12831. 37902119

[jbg70042-bib-0024] Guo, X. , G. Su , O. F. Christensen , L. Janss , and M. S. Lund . 2016. “Genome‐Wide Association Analyses Using a Bayesian Approach for Litter Size and Piglet Mortality in Danish Landrace and Yorkshire Pigs.” BMC Genomics 17: 468. 10.1186/s12864-016-2806-z. 27317562 PMC4912826

[jbg70042-bib-0025] Gustafsson, K. , G. Heffner , P. L. Wenzel , et al. 2013. “The Src Homology 2 Protein Shb Promotes Cell Cycle Progression in Murine Hematopoietic Stem Cells by Regulation of Focal Adhesion Kinase Activity.” Experimental Cell Research 319, no. 12: 1852–1864. 23528453 10.1016/j.yexcr.2013.03.020

[jbg70042-bib-0095] Halevy, A. 2014. “Novel EXOSC3 Mutation Causes Complicated Hereditary Spastic Paraplegia.” Journal of Neurology 261: 2165–2169. 10.1007/s00415-014-7457-x. 25149867

[jbg70042-bib-0026] Holm, B. , M. Bakken , G. Klemetsdal , and O. Vangen . 2004. “Genetic Correlations Between Reproduction and Production Traits in Swine.” Journal of Animal Science 82, no. 12: 3458–3464. 10.2527/2004.82123458x. 15537764

[jbg70042-bib-0027] Hong, Y. , C. Tan , X. He , et al. 2024. “Genome‐Wide Association Study of Reproductive Traits in Large White Pigs.” Animals 14, no. 19: 2874. 10.3390/ani14192874. 39409823 PMC11475698

[jbg70042-bib-0028] Hsu, W. L. , and R. K. Johnson . 2014. “Analysis of 28 Generations of Selection for Reproduction, Growth, and Carcass Traits in Swine.” Journal of Animal Science 92, no. 11: 4806–4822. 10.2527/jas.2014-8125. 25349336

[jbg70042-bib-0029] Jiang, Y. , S. Tang , W. Xiao , et al. 2019. “A Genome‐Wide Association Study of Reproduction Traits in Four Pig Populations With Different Genetic Backgrounds.” Asian‐Australasian Journal of Animal Sciences 33, no. 9: 1400–1410. 10.5713/ajas.19.0411. 32054232 PMC7468174

[jbg70042-bib-0030] Johnson, J. S. , K. R. Stewart , T. J. Safranski , J. W. Ross , and L. H. Baumgard . 2020. “In Utero Heat Stress Alters Postnatal Phenotypes in Swine.” Theriogenology 154: 110–119. 10.1016/j.theriogenology.2020.05.013. 32540511

[jbg70042-bib-0031] Kim, H. Y. , and S. Mohan . 2013. “Role and Mechanisms of Actions of Thyroid Hormone on the Skeletal Development.” Bone Research 1: 146–161. 10.4248/BR201302004. 26273499 PMC4472099

[jbg70042-bib-0032] Kinsella, R. J. , A. Kähäri , S. Haider , et al. 2011. “Ensembl BioMarts: A Hub for Data Retrieval Across Taxonomic Space.” Database: The Journal of Biological Databases and Curation 2011: 1–9. 10.1093/database/bar030. PMC3170168 21785142

[jbg70042-bib-0033] Kobek‐Kjeldager, C. , V. A. Moustsen , P. K. Theil , and L. J. Pedersen . 2020. “Effect of Litter Size, Milk Replacer and Housing on Production Results of Hyper‐Prolific Sows.” Animal 14, no. 4: 824–833. 10.1017/S175173111900260X. 31650940

[jbg70042-bib-0034] Kramer, L. M. , L. . M. Kramer , A. Wolc , et al. 2021. “Purebred‐Crossbred Genetic Parameters for Reproductive Traits in Swine.” Journal of Animal Science 99, no. 10: skab270. 10.1093/jas/skab270. 34558614 PMC8557628

[jbg70042-bib-0035] Kriz, V. , J. Mares , P. Wentzel , et al. 2007. “Shb Null Allele Is Inherited With a Transmission Ratio Distortion and Causes Reduced Viability In Utero.” Developmental Dynamics 236: 2485–2492. 10.1002/dvdy.21257. 17676633

[jbg70042-bib-0097] Li, J. , L. H. Geraldo , A. Dubrac , G. Zarkada , and A. Eichmann . 2021. “Slit2‐Robo Signaling Promotes Glomerular Vascularization and Nephron Development.” Journal of the American Society of Nephrology 32, no. 9: 2255–2272. 10.1681/ASN.2020111640. 34341180 PMC8729857

[jbg70042-bib-0036] Lian, W. , D. Gao , C. Huang , Q. Zhong , R. Hua , and M. Lei . 2022. “Heat Stress Impairs Maternal Endometrial Integrity and Results in Embryo Implantation Failure by Regulating Transport‐Related Gene Expression in Tongcheng Pigs.” Biomolecules 12: 388. 10.3390/biom12030388. 35327580 PMC8945854

[jbg70042-bib-0037] Liu, K. , L. Hou , Y. Yin , et al. 2023. “Genome‐Wide Association Study Reveals New QTL and Functional Candidate Genes for the Number of Ribs and Carcass Length in Pigs.” Animal Genetics 54, no. 4: 435–445. 10.1111/age.13315. 36911996

[jbg70042-bib-0038] Liu, Z. , H. Li , Z. Zhong , et al. 2022. “A Whole Genome Sequencing‐Based Genome‐Wide Association Study Reveals the Potential Associations of Teat Number in Qingping Pigs.” Animals 12, no. 9: 1058. 10.3390/ani12091057. 35565484 PMC9100799

[jbg70042-bib-0039] Lopez, B. I. , T. H. Kim , M. T. Makumbe , C. W. Song , and K. S. Seo . 2017. “Variance Components Estimation for Farrowing Traits of Three Purebred Pigs in Korea.” Asian‐Australasian Journal of Animal Sciences 30, no. 9: 1239–1244. 10.5713/ajas.17.0002. 28335089 PMC5582279

[jbg70042-bib-0040] Loueva, I. , M. Dauncey , J. Dividich , et al. 2000. “Regulation of Development by Nutrition and by the Somatotrophic and Thyroid Axes in the Neonatal Pig.” Livestock Production Science 66, no. 2: 121–131. 10.1016/S0301-6226(00)00219-0.

[jbg70042-bib-0041] Lourenco, D. , S. Tsuruta , I. Aguilar , et al. 2022. “Recent Updates in the BLUPF90 Software Suite. In Proceedings of 12th World Congress on Genetics Applied to Livestock Production. 1530–1533.” 10.3920/978-90-8686-940-4_366.

[jbg70042-bib-0042] Lu, X. , I. M. Abdalla , M. Nazar , et al. 2021. “Genome‐Wide Association Study on Reproduction‐Related Body‐Shape Traits of Chinese Holstein Cows.” Animals 11, no. 7: 1927. 10.3390/ani11071927. 34203505 PMC8300307

[jbg70042-bib-0043] Lunney, J. K. , A. Van Goor , K. E. Walker , T. Hailstock , J. Franklin , and C. Dai . 2021. “Importance of the Pig as a Human Biomedical Model.” Science Translational Medicine 13, no. 621: abd5758. 10.1126/scitranslmed.abd5758. 34818055

[jbg70042-bib-0044] Ma, X. , P. Li , Q. Zhang , et al. 2019. “Transcriptome Analysis of the Endometrium From Chinese Erhualian Sows That Differ in Calcium Ion Concentration and Litter Size.” Animal Genetics 50, no. 5: 542–550. 10.1111/age.12788. 31058330

[jbg70042-bib-0098] Mackay, D. 2006. “The Mouse ovol2 Gene Is Required for Cranial Neural Tube Development.” Developmental Biology 291, no. 1: 38–52. 10.1016/j.ydbio.2005.12.003. 16423343 PMC2891516

[jbg70042-bib-0045] Martins, T. F. , A. F. Braga Magalhães , L. L. Verardo , et al. 2022. “Functional Analysis of Litter Size and Number of Teats in Pigs: From GWAS to Post‐GWAS.” Theriogenology 193: 157–166. 10.1016/j.theriogenology.2022.09.005. 36209572

[jbg70042-bib-0046] Mazloomi, S. , M. Sanoeei Farimani , H. Tayebinia , et al. 2022. “The Association of Mitochondrial Translocator Protein and Voltage‐Dependent Anion Channel‐1 in Granulosa Cells With Estradiol Levels and Presence of Immature Follicles in Polycystic Ovary Syndrome.” Journal of Reproduction & Infertility 23, no. 3: 148–159. 10.18502/jri.v23i3.10005. 36415496 PMC9666595

[jbg70042-bib-0047] Mccauley, B. S. , and W. Dang . 2021. “Loosening Chromatin and Dysregulated Transcription: A Perspective on Cryptic Transcription During Mammalian Aging.” Briefings in Functional Genomics 21, no. 1: 56–61. 10.1093/bfgp/elab026. PMC8789305 34050364

[jbg70042-bib-0048] Momb, J. , J. P. Lewandowski , J. D. Bryant , et al. 2012. “Deletion of Mthfd1l Causes Embryonic Lethality and Neural Tube and Craniofacial Defects in Mice.” Proceedings of the National Academy of Sciences 110, no. 2: 549–554. 10.1073/pnas.1211199110. PMC3545794 23267094

[jbg70042-bib-0049] Moreira, G. C. M. , C. Boschiero , A. S. M. Cesar , et al. 2018. “A Genome‐Wide Association Study Reveals Novel Genomic Regions and Positional Candidate Genes for Fat Deposition in Broiler Chickens.” BMC Genomics 19: 374. 10.1186/s12864-018-4779-6. 29783939 PMC5963092

[jbg70042-bib-0050] Muñoz, G. , C. Ovilo , J. Estellé , L. Silió , A. Fernández , and C. Rodriguez . 2007. “Association With Litter Size of New Polymorphisms on ESR1 and ESR2 Genes in a Chinese‐European Pig Line.” Genetics Selection Evolution 39: 195–206. 10.1186/1297-9686-39-2-195. PMC2682837 17306201

[jbg70042-bib-0051] Neil, H. , C. Malabat , Y. d'Aubenton‐Carafa , et al. 2009. “Widespread Bidirectional Promoters Are the Major Source of Cryptic Transcripts in Yeast.” Nature 457: 1038–1042. 10.1038/nature07747. 19169244

[jbg70042-bib-0099] Ng, A. Q. E. , S. N. Chan , and J. W. Pek . 2024. “Nutrient‐Dependent Regulation of a Stable Intron Modulates Germline Mitochondrial Quality Control.” Nature Commun. 10.1038/s41467-024-45651-y. PMC10858910 38341415

[jbg70042-bib-0052] Nielsen, B. , G. Su , M. S. Lund , et al. 2013. “Selection for Increased Number of Piglets at d 5 After Farrowing Has Increased Litter Size and Reduced Piglet Mortality.” Journal of Animal Science 91, no. 6: 2575–2582. 10.2527/jas.2012-5990. 23508021

[jbg70042-bib-0053] Ogawa, S. , A. Konta , M. Kimata , et al. 2018. “Estimation of Genetic Parameters for Farrowing Traits in Purebred Landrace and Large White Pigs.” Animal Science Journal 90, no. 1: 23–28. 10.1111/asj.13120. 30370591 PMC6587850

[jbg70042-bib-0054] Ogawa, S. , C. Ohnishi , K. Ishii , et al. 2020. “Genetic Relationship Between Litter Size Traits at Birth and Body Measurement and Production Traits in Purebred Duroc Pigs.” Animal Science Journal 91, no. 1: e13497. 10.1111/asj.13497. 33368835

[jbg70042-bib-0055] Onteru, S. K. , D. M. Gorbach , J. M. Young , D. J. Garrick , J. C. Dekkers , and M. F. Rothschild . 2013. “Whole Genome Association Studies of Residual Feed Intake and Related Traits in the Pig.” PLoS One 8: 1756. 10.1371/journal.pone.0061756. PMC3694077 23840294

[jbg70042-bib-0056] Osellame, L. D. 2012. “Cellular and Molecular Mechanisms of Mitochondrial Function.” Best Practice & Research Clinical Endocrinology & Metabolism 26, no. 6: 711–723. 10.1016/j.beem.2012.05.003. 23168274 PMC3513836

[jbg70042-bib-0057] Padilha, S. F. , R. Martins , L. M. Hul , et al. 2025. “Genome‐Wide Association Analysis Reveals Insights Into the Genetic Architecture of Mesenteric Torsion in Pigs.” Scientific Reports 15, no. 1: 13774. 10.1038/s41598-025-98029-5. 40258920 PMC12012111

[jbg70042-bib-0058] Paterni, I. , C. Granchi , J. A. Katzenellenbogen , et al. 2014. “Estrogen Receptors Alpha (ERα) and Beta (ERβ): Subtype‐Selective Ligands and Clinical Potential.” Steroids 90: 13–29. 10.1016/j.steroids.2014.06.012. 24971815 PMC4192010

[jbg70042-bib-0059] Pierzchała, M. , D. Pierzchała , M. Ogłuszka , et al. 2020. “Identification of Differentially Expressed Gene Transcripts in Porcine Endometrium During Early Stages of Pregnancy.” Life 10, no. 5: 68. 10.3390/life10050068. 32429378 PMC7281126

[jbg70042-bib-0060] Putz, A. M. , F. Tiezzi , C. Maltecca , et al. 2015. “Variance Component Estimates for Alternative Litter Size Traits in Swine.” Journal of Animal Science 93, no. 11: 5153–5163. 10.2527/jas.2015-9416. 26641035

[jbg70042-bib-0061] Roehe, R. , and B. W. Kennedy . 1995. “Estimation of Genetic Parameters for Litter Size in Canadian Yorkshire and Landrace Swine With Each Parity of Farrowing Treated as a Different Trait.” Journal of Animal Science 73, no. 10: 2959. 10.2527/1995.73102959x. 8617666

[jbg70042-bib-0062] Ross, J. W. , B. J. Hale , J. T. Seibert , et al. 2017. “Physiological Mechanisms Through Which Heat Stress Compromises Reproduction in Pigs.” Molecular Reproduction and Development 84, no. 9: 934–945. 10.1002/mrd.22859. 28667793

[jbg70042-bib-0063] Rothchild, M. , C. Jacobson , D. Vaske , et al. 1996. “The Estrogen Receptor Locus Is Associated With a Major Gene Influencing Litter Size in Pigs.” Proceedings of the National Academy of Science 93, no. 1: 201–205. 10.1073/pnas.93.1.20. PMC40206 8552604

[jbg70042-bib-0064] Sayers, E. W. , E. E. Bolton , J. R. Brister , et al. 2021. “Database Resources of the National Center for Biotechnology Information.” Nucleic Acids Research 1: D20–D26. 10.1093/nar/gkab1112. PMC8728269 34850941

[jbg70042-bib-0065] Schneider, J. F. , J. R. Miles , T. M. Brown‐Brandl , J. A. Nienaber , G. A. Rohrer , and J. L. Vallet . 2015. “Genome‐Wide Association Analysis for Average Birth Interval and Stillbirth in Swine.” Journal of Animal Science 93, no. 2: 529–540. 10.2527/jas.2014-7899. 26020742

[jbg70042-bib-0066] Sell‐Kubiak, E. 2021. “Selection for Litter Size and Litter Birthweight in Large White Pigs: Maximum, Mean and Variability of Reproduction Traits.” Animal 15, no. 10: 100352. 10.1016/j.animal.2021.100352. 34534762

[jbg70042-bib-0067] Sell‐Kubiak, E. , E. F. Knol , and M. Lopes . 2022. “Evaluation of the Phenotypic and Genomic Background of Variability Based on Litter Size of Large White Pigs.” Genetics Selection Evolution 54: 1–15. 10.1186/s12711-021-00692-5. PMC8722267 34979897

[jbg70042-bib-0068] Shin, M. , A. Vaughn , J. Momb , et al. 2019. “Deletion of Neural Tube Defect‐Associated Gene Mthfd1l Causes Reduced Cranial Mesenchyme Density.” Birth Defects Research 111, no. 18: 1202–1209. 10.1002/bdr2.1591. PMC6939463 31518072

[jbg70042-bib-0069] Silva, J. F. , N. M. Ocarino , R. Serakides , et al. 2018. “Thyroid Hormones and Female Reproduction.” Biology of Reproduction 99, no. 5: 907–921. 10.1093/biolre/ioy115. 29767691

[jbg70042-bib-0070] Su, G. 2020. “Possibilities for Genetic Improvement of Pig Survival Until Slaughter. Tjele DCA—National Centre for Food and Agriculture, Aarhus University.” https://pure.au.dk/ws/portalfiles/portal/207547908/Levering_H_jere_overlevelse_for_pattegrise_og_sm_grise_fra_dag_5_indtil_slagtning_gennem_kvantitativ_avl.pdf.

[jbg70042-bib-0071] Su, G. , D. Sorensen , and M. S. Lund . 2007. “Selection for Litter Size at Day Five to Improve Litter Size at Weaning and Piglet Survival Rate.” Journal of Animal Science 85, no. 6: 1385–1392. 10.2527/jas.2006-631. 17339413

[jbg70042-bib-0072] Su, G. , D. Sorensen , M. S. Lund , et al. 2008. “Variance and Covariance Components for Liability of Piglet Survival During Different Periods.” Animal 2, no. 2: 184–189. 10.1017/S1751731107001115. 22445011

[jbg70042-bib-0100] Sun, J. , J. Xiao , Y. Jiang , et al. 2023. “Genome‐Wide Association Study on Reproductive Traits Using Imputation‐Based Whole‐Genome Sequence Data in Yorkshire Pigs.” Genes 2, no. 14: 861. 10.3390/genes14040861. PMC10137786 37107619

[jbg70042-bib-0073] Supek, F. , M. Bošnjak , N. Škunca , and T. Šmuc . 2011. “REVIGO Summarizes and Visualizes Long Lists of Gene Ontology Terms.” PLoS One 6, no. 7: e21800. 10.1371/journal.pone.0021800. 21789182 PMC3138752

[jbg70042-bib-0074] Szklarczyk, D. , A. L. Gable , D. Lyon , et al. 2019. “STRING v11: Protein‐Protein Association Networks With Increased Coverage, Supporting Functional Discovery in Genome‐Wide Experimental Datasets.” Nucleic Acids Research 8, no. 47: D607–D613. 10.1093/nar/gky1131. PMC6323986 30476243

[jbg70042-bib-0075] Tang, Z. , J. Xu , L. Yin , et al. 2019. “Genome‐Wide Association Study Reveals Candidate Genes for Growth Relevant Traits in Pigs.” Frontiers in Genetics 10: 02. 10.3389/fgene.2019.00302. PMC6459934 31024621

[jbg70042-bib-0076] Thomas, P. D. 2021. “PANTHER: Making Genome‐Scale Phylogenetics Accessible to All.” Protein Science 31: 8–22. 10.1093/nar/gky1131. 34717010 PMC8740835

[jbg70042-bib-0077] Unezaki, S. , R. Horai , K. Sudo , Y. Iwakura , and S. Ito . 2007. “Ovol2/Movo, a Homologue of Drosophila Ovo, Is Required for Angiogenesis, Heart Formation and Placental Development in Mice.” Genes to Cells 12: 773–785. 10.1111/j.1365-2443.2007.01084.x. 17573777

[jbg70042-bib-0078] Uzzaman, M. R. , J. E. Park , K. T. Lee , E. S. Cho , B. H. Choi , and T. H. Kim . 2018. “A Genome‐Wide Association Study of Reproductive Traits in a Yorkshire Pig Population.” Livestock Science 209: 67–72. 10.1016/j.livsci.2018.01.005.

[jbg70042-bib-0079] van der Werf, J. 2003. “Chapter 18: Multiple Trait Genetic Evaluation.” In Materials on Linear Models in Animal Breeding, edited by J. van der Werf . University of New England. https://jvanderw.une.edu.au/.

[jbg70042-bib-0080] VanRaden, P. M. 2007. “Genomic Measures of Relationship and Inbreeding. Animal Improvement Programs Laboratory, Agricultural Research Service, United States Department of Agriculture, Beltsville, MD, USA. Proceedings of the 2007 Interbull Meeting, 37.”

[jbg70042-bib-0102] Wang, H. , I. Misztal , I. Aguilar , A. Legarra , and W. M. Muir . 2012. “Genome‐Wide Association Mapping Including Phenotypes from Relatives Without Genotypes.” Genetics Research 94, no. 2: 73–83. 10.1017/s0016672312000274. 22624567

[jbg70042-bib-0081] Wu, Z. C. , Y. Wang , X. Huang , et al. 2022. “A Genome‐Wide Association Study of Important Reproduction Traits in Large White Pigs.” Gene 838: 146702. 10.1016/j.gene.2022.146702. 35772658

[jbg70042-bib-0082] Xu, Z. , H. Sun , Z. Zhang , et al. 2019. “Selection Signature Reveals Genes Associated With Susceptibility Loci Affecting Respiratory Disease due to Pleiotropic and Hitchhiking Effect in Chinese Indigenous Pigs.” Asian‐Australasian Journal of Animal Sciences 33, no. 2: 187–196. 10.5713/ajas.18.0658. 30744329 PMC6946968

[jbg70042-bib-0083] Yang, H. , Y. M. Yoo , E. M. Jung , et al. 2010. “Uterine Expression of Sodium/Potassium/Calcium Exchanger 3 and Its Regulation by Sex‐Steroid Hormones During the Estrous Cycle of Rats.” Molecular Reproduction and Development 77, no. 10: 913–920. 10.1002/mrd.21245. 21104767

[jbg70042-bib-0084] Yang, Y. , M. Gan , X. Yang , et al. 2023. “Estimation of Genetic Parameters of Pig Reproductive Traits.” Frontiers in Veterinary Science 10: 2287. 10.3389/fvets.2023.1172287. PMC10321596 37415962

[jbg70042-bib-0085] Yu, G. , C. Wang , and Y. Wang . 2022. “Genetic Parameter Analysis of Reproductive Traits in Large White Pigs.” Animal Bioscience 35, no. 11: 1649–1655. 10.5713/ab.22.0119. 36108704 PMC9659455

[jbg70042-bib-0086] Zaalberg, R. M. , T. T. Chu , H. Bovbjerg , J. Jensen , and T. M. Villumsen . 2023. “Genetic Parameters for Early Piglet Weight, Litter Traits, and Number of Functional Teats in Organic Pigs.” Animal 17, no. 3: 100717. 10.1016/j.animal.2023.100717. 36791491

[jbg70042-bib-0087] Zhang, H. , Z. Liu , J. Wang , T. Zeng , X. Ai , and K. Wu . 2023. “An Integrative ATAC‐Seq and RNA‐Seq Analysis of the Endometrial Tissues of Meishan and Duroc Pigs.” International Journal of Molecular Sciences 24, no. 19: 14812. 10.3390/ijms241914812. 37834260 PMC10573446

[jbg70042-bib-0088] Zhang, R. , F. Yao , X. Cheng , M. Yang , and Z. Ning . 2023. “Identification of Candidate Genomic Regions for Egg Yolk Moisture Content Based on a Genome‐Wide Association Study.” BMC Genomics 24: 110. 10.1186/s12864-023-09221-8. 36918797 PMC10015838

[jbg70042-bib-0089] Zhang, S. , J. Zhang , B. S. Olasege , et al. 2019. “Estimation of Genetic Parameters for Reproductive Traits in Connectedness Groups of Duroc, Landrace, and Yorkshire Pigs in China.” Journal of Animal Breeding and Genetics 137, no. 2: 211–222. 10.1111/jbg.12431. 31468579

[jbg70042-bib-0090] Zhang, T. , L. Wang , H. Shi , et al. 2015. “Heritabilities and Genetic and Phenotypic Correlations of Litter Uniformity and Litter Size in Large White Sows.” Journal of Animal Science and Biotechnology 6: 15. 10.1016/S2095-3119(15)61155-8.25937926

[jbg70042-bib-0091] Zhao, Y. X. , G. X. Gao , Y. Zhou , et al. 2022. “Genome‐Wide Association Studies Uncover Genes Associated With Litter Traits in the Pig.” Animal 16, no. 12: 100672. 10.1016/j.animal.2022.100672. 36410176

[jbg70042-bib-0092] Zhou, G. , O. Soufan , J. Ewald , R. E. W. Hancock , N. Basu , and J. Xia . 2019. “NetworkAnalyst 3.0: A Visual Analytics Platform for Comprehensive Gene Expression Profiling and Meta‐Analysis.” Nucleic Acids Research 2: W234–W241. 10.1093/nar/gkz240. PMC6602507 30931480

[jbg70042-bib-0093] Zhou, R. , Y.‐l. Yang , Y. Liu , J. Chen , B. Yang , and Z.‐l. Tang . 2023. “High Serum Reproductive Hormone Levels at Mid‐Pregnancy Support Meishan Pig Prolificacy.” Journal of Integrative Agriculture 22, no. 11: 3489–3499. 10.1016/j.jia.2023.05.014.

